# Pathway-based classification of cancer subtypes

**DOI:** 10.1186/1745-6150-7-21

**Published:** 2012-07-03

**Authors:** Shinuk Kim, Mark Kon, Charles DeLisi

**Affiliations:** 1 Bioinformatics program, Boston University, Boston, MA, 02215, USA; 2 Department of Mathematics and Statistics, Boston University, Boston, MA, 02215, USA; 3 Bioinformatics program, Boston University, 24 Cummington Street, Boston, MA, 02215, USA

**Keywords:** Classification, Pathway, Cancer subtypes, Ovarian cancer, Breast cancer, Gene set enrichment analysis

## Abstract

**Background:**

Molecular markers based on gene expression profiles have been used in experimental and clinical settings to distinguish cancerous tumors in stage, grade, survival time, metastasis, and drug sensitivity. However, most significant gene markers are unstable (not reproducible) among data sets. We introduce a standardized method for representing cancer markers as 2-level hierarchical feature vectors, with a basic gene level as well as a second level of (more stable) pathway markers, for the purpose of discriminating cancer subtypes. This extends standard gene expression arrays with new pathway-level activation features obtained directly from off-the-shelf gene set enrichment algorithms such as GSEA. Such so-called pathway-based expression arrays are significantly more reproducible across datasets. Such reproducibility will be important for clinical usefulness of genomic markers, and augment currently accepted cancer classification protocols.

**Results:**

The present method produced more stable (reproducible) pathway-based markers for discriminating breast cancer metastasis and ovarian cancer survival time. Between two datasets for breast cancer metastasis, the intersection of standard significant gene biomarkers totaled 7.47% of selected genes, compared to 17.65% using pathway-based markers; the corresponding percentages for ovarian cancer datasets were 20.65% and 33.33% respectively. Three pathways, consisting of Type_1_diabetes mellitus, Cytokine-cytokine_receptor_interaction and Hedgehog_signaling (all previously implicated in cancer), are enriched in both the ovarian long survival and breast non-metastasis groups. In addition, integrating pathway and gene information, we identified five (ID4, ANXA4, CXCL9, MYLK, FBXL7) and six (SQLE, E2F1, PTTG1, TSTA3, BUB1B, MAD2L1) known cancer genes significant for ovarian and breast cancer respectively.

**Conclusions:**

Standardizing the analysis of genomic data in the process of cancer staging, classification and analysis is important as it has implications for both pre-clinical as well as clinical studies. The paradigm of diagnosis and prediction using pathway-based biomarkers as features can be an important part of the process of biomarker-based cancer analysis, and the resulting canonical (clinically reproducible) biomarkers can be important in standardizing genomic data. We expect that identification of such canonical biomarkers will improve clinical utility of high-throughput datasets for diagnostic and prognostic applications.

**Reviewers:**

This article was reviewed by John McDonald (nominated by I. King Jordon), Eugene Koonin, Nathan Bowen (nominated by I. King Jordon), and Ekaterina Kotelnikova (nominated by Mikhail Gelfand).

## Background

The identification of genome-wide expression profiles that discriminate between disease phenotypes is now a relatively routine research procedure. However, clinical implementation has been slow, in part because marker sets identified by independent studies rarely display substantial overlap [1-4]. For example, van't Veer *et al. *[4], and Wang *et al. *[3] identified gene sets of size 70 and 76 to distinguish metastatic from non-metastatic breast cancer, but the two sets had an overlap of only 3 genes [5]. As another example, Dressman *et al. *[2] found a set of 100 genes significant in predicting responses to cis-platinum therapy for ovarian cancer using a stochastic search method, which had no intersection with another set of 86 genes predictive of ovarian survival found by Crijns *et al. *[1] using functional class scoring analysis. Though the latter pair of sets was not studied to predict identical phenotypes in ovarian cancer, their empty intersection still indicates the lack of uniformity in predictive biomarkers among different experiments.

There is a well-recognized need for *canonical* biomarkers, which will make it possible to record marker-based data interchangeably among different laboratories, using well-recognized quantitative features to encode cancer and other phenotypes. This need is related to future improvements in standardized diagnosis and prognosis regimes which will incorporate genomic cancer information as a matter of course, augmenting current cancer classification protocols.

In connection with this, many researchers have suggested a more effective and robust means of marker identification which combines gene expression measurements over functional or otherwise naturally defined sets of genes. For example, Chuang *et al. *[5] used average differential expressions in protein-protein interaction subnetworks as markers for distinguishing metastatic from non-metastatic breast cancer using the Wang *et al. *[3] and van de Vijver *et al. *[6] data sets. The stability (overlap) between the two independent datasets of their selected critical genes is 12.7%, whereas it is 7% for critical individual gene markers. This overlap was calculated between 906 and 618 critical genes obtained from the Wang and van de Vijver data sets, respectively [5]. However, protein-protein networks do not yield canonical coherent gene subsets as pathways do, and such critical gene sets change with individual experiments. In this regard, pathways, being the most documented of protein interactions, yield stable sets of functional relationships related with molecular biological activities such as metabolic, signaling, protein interaction and gene regulation processes [7]. Nevertheless, pathway-based aggregation of gene information is one among a number of ways of incorporating gene-gene relationships in augmenting predictive performance, and protein-protein interaction-based as well as coexpression-based aggregations have been shown in various contexts to improve performance of classification methods [5].

What distinguishes this work from the above is our goal to obtain collections of biomarkers which are not only discriminative between phenotypes, but are also canonical, in that they come from standardized gene sets in the form of pathways and other functional sets. This in turn fits into a view toward clinical applications in which comprehensible and reproducible biomarkers can be extracted and used for phenotype prediction, using existing standardized gene set enrichment algorithms.

With the recent availability of large quantities of pathway information such as KEGG (Ogata *et al.*, 1999), GeneGo (http://www.genego.com) and BioCarta (http://www.biocarta.com[8]), pathway-based analysis has been used [9-11] to perform classification of expression profiles and also applied to discriminate different classes of disease. Class distinction based on differences in pathway activity can be more stable than distinction based on genes alone. For instance, 16 pathways overlapped between the 48 significant pathways obtained from the study of Dressman *et al. *[2] and the 17 pathways from Crijns *et al. *[1], using the methodology in Crijns *et al.* for significant pathway identification. Thus, pathway markers (and perhaps other gene set markers) are more reproducible than individual genes selected from expression profiles. A growing body of research has focused on pathway-based classification, and has often presented comparable or better performance of classification than gene-based classifiers [9-11]. For example, Guo *et al. *[9] proposed the use of mean or median gene expression values in gene ontology (GO) modules [12] to infer module activity. Recently, Su *et al. *[11] proposed a classification method based on probabilistic inference of pathway activity.

Other pathway-level analyses which have led to classification methods for cancer phenotypes include the work of Lee *et al. *[10], who identified core genes in pathways as differentiators of disease phenotypes. Vaske *et al. *[13] inferred pathway-level perturbations in cancer tissue based on omics-level analyses of individual genes using a factor graph model, yielding inferences regarding survival outcomes. Breslin *et al. *[14] used pathway signatures based on activations of their downstream genes to classify cancer samples. Svensson *et al. *[15] used pathway signatures to differentiate radiation toxicity responses in irradiated tissues involved in the treatment of prostate cancer.

The above uses of pathway-level inference to classify phenotypes provide evidence of the usefulness of better and more stable (reproducible) biomarkers related to gene expression measurements. In this paper we focus on measuring the stability of pathway-based biomarkers and evaluating protocols for using standardized (as well as some new) gene set enrichment algorithms to directly and automatically infer pathway activation levels as stable features able to discriminate cancer phenotypes. This can provide methods for supplementing or replacing gene-level activation features with additional features which form new biomarkers based directly on pathway-level activations.

Our use of the term pathway activation (or activity) markers parallels usage in other references [10,14] and denotes transcriptional activity of genes in these pathways which act coherently within them. In particular this term is not used in the biological sense that the pathway has been activated into producing downstream products, which generally may be the case in one of its differentiating (e.g. case vs. control) states.

The result of precisely quantifying such pathway-level activity can be new standardized sets of biomarkers (‘pathway-based expression arrays’) which we will show to be consistently more reproducible across different data sets. We study the stability properties of these biomarkers, in addition to their discriminatory abilities among phenotypes. A desired application of these results involves eventual clinical uses which will be capable of using off the shelf enrichment algorithms for genes (e.g. GSEA, [16]) to automatically infer phenotype differences based on pathway (and other gene set) -level feature vectors.

The examples in this paper use RNA expression arrays from microarrays. However, current new methods of obtaining mRNA expression levels from RNA-Seq abundances (using methods such as Cufflinks [17] and NEUMA [18]) can also produce gene-level (mRNA) expression arrays which can be analyzed in the same way using the pathway-aggregated methods (e.g. GSEA and related algorithms) discussed in this paper. In addition, with newer RNA-Seq data, other groups of biologically related RNA transcripts may also be candidates for aggregation of individual RNA abundance levels in the same way as is done here for mRNA levels of pathway genes.

In this paper we obtain stable mRNA expression-based markers at two levels, the pathway level and the gene level. Given a stable pathway *P* involved in a given cancer phenotype, we can also identify stable gene sets based on *P*, by identifying the most discriminative genes in *P*, again using a standard enrichment algorithm such as GSEA. (In the latter case the discriminative genes can be identified by finding the leading edge genes in *P*).

We remark that the use of multi-level hierarchical feature aggregates, with individual genes at the first level and initial gene aggregates (pathways) at the next level, is an instance of a machine learning approach which organizes individual features into a *hierarchical feature structure*. The application of an SVM classifier to features derived from such a structure (as is done here) is known as a *hierarchical SVM.* A general hierarchical feature structure organizes individual features *x*_*i*_ in a feature vector x = (*x*_1_,…, *x*_*n*_) into a tree hierarchy. In such a hierarchy the first level (e.g., gene-level) features form the leaves of the tree, with aggregate features forming the second level, and higher order aggregates recursively forming the upper structure of the tree. A more general example of such a structure (than the present pathway-based hierarchy) involves, for a given gene set, a tree structure based on a gene ontology (GO) [12] tree, which will be studied in future work.

We remark in addition that since our primary purpose here is to develop protocols involving canonical biomarkers (markers obtained using standard methodologies such as GSEA that are also stable across datasets and platforms) for identifying cancer phenotypes, the strategy of using pathway-level aggregate features would require focusing on biomarkers within a given pathway *P*, even if there are better markers outside of this or perhaps any other pathway. A benefit of this is indeed that such additional associated genes in *P* would in fact be 'false negatives,' since they are ostensibly weak classifiers which nevertheless cooperate with genes in a pathway *P* that has had its role in the phenotype established.

Gene set enrichment analysis (GSEA) proposed by Subramanian *et al. *[16] and extended by Hung *et al. *[19] to include network topology is a useful tool for pathway-based class distinction. Briefly, GSEA determines whether genes from a particular pathway or some other predefined gene set are significantly overrepresented in the set of genes that are most differentially expressed. Since the differentially expressed genes are rank ordered, the procedure also returns so-called leading edge genes for which enrichment has its maximum value. The leading edge genes of a given pathway are those genes which as a group maximally differentiate the gene expression signatures (in the pathway) of the two classes being studied [16].

The approach in this paper is to use prior classes of gene groups (e.g., KEGG pathways, functional gene sets from the Molecular Signatures Databases; MSigDB [16]) to identify group-based biomarkers which can reliably and reproducibly classify medical and biological samples. We will show this using classifications based on the 200 curated KEGG pathways and 522 functional gene sets from MSigDB [16] collected from eight data sets and experimental literature, and compare these with classifications based on direct gene expression profiles. This methodology can provide (pathway-based) biomarkers with significantly increased stability for differentiating cancer as well as other phenotypes, as is shown here for ovarian and breast cancer (see results and discussion). In addition to the primary aim of greatly increased marker stability, the predictive accuracy using these biomarkers shows overall improvement (again for survival and metastasis prediction).

The pathway-based biomarkers related to ovarian cancer survival time as well as to breast cancer metastasis yield important stable pathways in both types of datasets (see below), most of which have had independently demonstrated involvement in cancer. In addition, we identify stable pathways between studies on breast cancer on the one hand and ovarian cancer on the other (the cytokine-cytokine receptor interaction, type 1 diabetes mellitus, and hedgehog signaling pathways), all three of which have been demonstrated in a number of independent studies to have significant involvement in cancer (see Results/Discussion). For the diabetes mellitus pathway in particular, the cancer-related immune system arm (involving the HLA family of genes) is the only portion capable of differentiating survival time in ovarian cancer or in determining metastasis in breast cancer.

Ovarian cancer is the fifth leading cause of death from gynecological malignancy in the United States and Western Europe [20]. The major histological subtypes are classified as mucinous, serous, endometrioid, and clear cell. Among these, serous carcinoma is the most common ovarian cancer subtype [21]. Carcinoma of the ovary is subclassified into 4 tumor stages defined by how that disease has spread (metastasized) from the original site to other parts of the body. The guidelines defining stages are provided from the International Federation of Gynecology and Obstetrics (FIGO). In particular, in stage IV, cancer has spread beyond the abdomen into tissues in the liver and other organs (NCI, FIGO). Some 75% of all patients with ovarian cancer are commonly diagnosed with stage III/IV disease, for which the 5-year survival rate is only 5% - 30%, with 21 months being the average survival time [1]. Consequently, a convenient diagnostic for early stage disease [1,2] that could be routinely applied has the potential to prolong survival by enabling early intervention. Similarly, the identification of biomarkers that would distinguish short from long term survivors who are on the same therapeutic regimen can help guide therapy.

Similarly, breast cancer has a large impact on health, being the primary cancer which strikes women overwhelmingly, and the second leading cause of cancer death in women. There is high variability in the outcomes and responses to therapies among patients in the same stage of this disease [22,23]. Though hormonal and chemo-therapies can significantly reduce the chances of metastases in this disease [24], predictions of such outcomes based on genomic information have not been implemented in a standardizable way.

Identification of biomarkers differentiating stages or expected survival terms in ovarian cancer, as well as metastasis in breast cancer, can help guide treatment. In this paper, we suggest our standardized pathway-based approach to biomarker analysis for discriminating cancer subtypes as a research tool with strong potential for clinical applications. The methodologies in this paper are validated in the identification of specific stable pathway markers (see below) which differentiate cancer phenotypes and validate previous studies identifying the same pathways and their component genes as significantly linked to cancer.

## Methods

### Materials

We used gene expression profiles from The Cancer Genome Atlas (TCGA; available at http://cancergenome.nih.gov/) for classification of ovarian phenotypes based on ovarian cancer tissues. The first ovarian cancer data set was generated by the Broad Institute (BI) using an HT_HG_U133A platform, with 287 cancer samples with expression measurements of 12,042 genes. The second dataset was obtained from the University of North Carolina (UNC) using a different Agilent G4502A_07 platform, and different samples compiled from the same 287 subjects but separate tissue samples, with measurements of 17,814 genes. The TCGA secured clinical information provides disease stage, initial diagnosis date and survival time of individual patients. We extracted gene expression profiles of 10 early stage (stage I and II) patients, and 49 late stage (IV) patients in both the BI and UNC datasets. In order to separate the samples into classes based on survival time from initial diagnosis, we separated the subjects into two groups consisting of those with survival times of less than 1 year with 22 samples (short survival) and greater than 5 years with 22 samples (long survival) from the total 287 samples. We used the gene expression data without initial gene filtering and compared the two data sets, with regard to the utility of pathway-based biomarkers in classification of the two classes. Further, we performed a similar analysis of classification of early stage vs. stage IV of ovarian cancer.

For biomarker discrimination tasks using a support vector machine (SVM) classifier, we used the Spider machine learning package, version 1.71 [25]. For all SVM tasks we used the Spider quadratic optimizer, with *C* parameter set to infinity. This choice of *C* is appropriate to high dimensional situations in which the degrees of freedom (i.e. number of genes) exceed the data size.

In addition, in order to validate the stability of the pathway biomarkers in comparison with single gene biomarkers, we also analyzed gene expression profiles from breast cancer metastasis data of Wang *et al. *[3] and van de Vijver *et al. *[6], with 286 and 295 breast cancer subjects respectively. For the Wang expression data sets, we converted an original 22,284 probe ID’s to 13,058 gene symbols using the Ailun package [26] and split the data set into two groups: one included 93 samples of cancers which metastasized within 5 years and the second included 183 samples of cancers which were not observed to metastasize within that time period. We used a total of 276 samples out of 286, since we eliminated 10 censored (incomplete data) patients (Wang *et al.*, [3]). In the van de Vijver data sets, we analyzed the gene expression data from 295 breast cancer subjects, including 79 who were diagnosed as metastatic within 5 years, and 216 non-metastatic samples. This data set covered 12,183 genes. For the pathway-based analysis, we analyzed gene groupings based on the 200 standard KEGG pathways (http://www.genome.jp/kegg/), and the 522 curated functional pathways from MSigDB [16]. In addition, we manually curated five ovarian cancer-related gene sets derived from five separate research studies (unrelated to the present studies); we denote these as the clear cell gene set [27], favorable prognosis gene set [1], platinum response gene set [2], ovarian cancer module 1 [28], and clear cell poor prognosis gene set [29].

### Methods

In order to perform some of the discriminations between cancer categories discussed in this paper, we utilized a methodological approach involving what are denoted here as *hierarchical feature vectors* in machine learning. These were used as input for our SVM classification algorithms. The use of such structured feature vectors in machine learning mirrors a categorization approach which is helpful in all learning processes, in which higher order (derived) features (concepts) are used along with elementary ones for sample classification. In general a hierarchical feature space vector method assigns to the features used in a classification task a hierarchical structure, in which basic features form the leaves of a tree, and higher order (derived) features form the internal nodes. In this case the raw features (leaves) are the gene expression features *x*_*i*_*,* while the derived features (which here are the only additional features used) are aggregate pathway features derived here. Though in this paper we consider only a two-level hierarchy, we anticipate that a more comprehensive use of levels may generally be useful. In particular, pathway categories may also be useful in this type of analysis for further stabilization and improvement of cancer classifications.

We examined this application of the hierarchical SVM, which creates higher-level biomarkers (generally more stable across different experiments) and improves classification by agglomerating gene information into pathway features. This resulted in an algorithm for discriminating sets of canonical pathway markers which were stable in distinguishing both early stage from late stage cancer, and short term survival from long term survival. These markers involve pathway activation measures obtained from the Gene Set Enrichment Algorithm (GSEA, Subramanian *et al.*, [16]) and an SVM-based pathway selection method (SPF, see method 3. below), both of which are determined by pathways containing genes which are the most up- and down-regulated between the classes to be separated.

**Figure 1:**
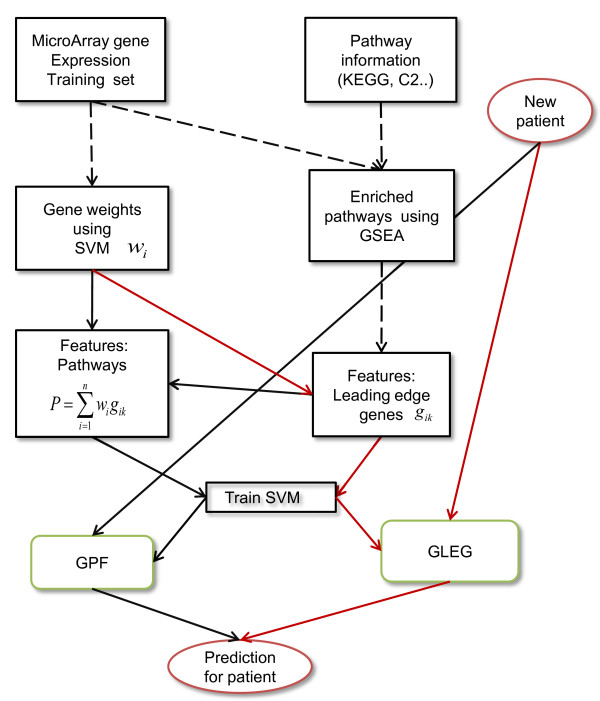
**Schematic diagram of the methods.** Black arrows represent the workflow of the GPF method, which used pathway features generated by combinations of GSEA leading edge genes and SVM gene weights. Red arrows represent the GLEG method, which directly classified using leading edge genes obtained from GSEA. Black dashed arrows represent workflow shared between GPF and GLEG. The SPF method used the same workflow as the GPF method with the replacement of pathway selections using GSEA by pathway selections using SVM. The *train SVM* step uses separate training features for the GPF and the GLEG workflows.

We introduce one approach for developing leading edge gene features determined by GSEA, and an additional two approaches for developing pathway features using GSEA and SVM (algorithm is available in Additional file 1: Table S1). A schematic diagram of the methods is presented in Figure 1.

1. **GLEG: using GSEA leading edge genes as features**

For any experiment involving differential gene expression data, pre-identified gene sets such as the KEGG pathways which are enriched in the most differentially expressed genes are scored and ranked using the GSEA algorithm (Subramanian *et al.*, [16]). The ranking is done by obtaining GSEA *p*-values for enrichment of a given pathway *P.* We denote the highest-ranking *l* pathways based on *p*-value as {*p*_1_,…, *p*_*l*_} (the latter notation is unrelated to the numerical *p* values used here), these being the most differentially expressed pathways between the two classes. For simplicity, with all data sets, we chose the 20 most up- and 20 most down-regulated (*enriched*) pathways, whose union formed a set of 40 classifying pathways, representing approximately 20% of all pathways used. Each enriched pathway contains a set of *leading edge* genes {*g*_1_,…, *g*_*k*_}, which form the most discriminatory genes in the pathway, as returned by the GSEA algorithm. GSEA determines leading edge genes by scoring a maximum running sum along the ranked list of differentially expressed genes.

The GLEG (GSEA-based Leading Edge Gene feature) method collects the leading edge genes in the above top GSEA 40 pathways, and uses them directly as features in an SVM to discriminate between the two classes of interest. The enriched pathways and their leading edge genes were learned as a reduced feature set using the training data sets, and on this feature set an SVM was trained and then tested on the test set. The number 40 (out of 200 total pathways) was chosen so that a nontrivial overlap between two groups of 40 pathways from two experiments could provide statistically reliable results. See the Results section for more information on the algorithm.

2. **GPF: using GSEA-enriched pathways as features**

In the GPF (GSEA Pathway Feature) approach, our discrimination features corresponded to the 40 pathways mentioned above (for each data set) which had the most differential expression in the training data. In training and testing, we computed each pathway feature (denoted *P*_*j*_ for pathway *j*) using a linear combination of the expression levels *g*_*ij*_ of the leading edge genes in pathway *j*, weighted by their SVM global weights *w*_*ij*_, inherited from weights in training data differentiating the two classes without any gene feature selection.

Specifically, the pathway features are

(1) $$P_{j}=\sum_{ij}w_{\mathrm{ij}}g_{\mathrm{ij}}$$

where *P*_*j*_ is the pathway activation level, *g*_*ij*_ is the gene expression level, and *w*_*ij*_ is the inherited SVM weight with indices of the *i*^*th*^ gene in the *j*^*th*^ pathway. The Results section describes the implementation of this algorithm in more detail.

3. **SPF: using pathway features determined by SVM**

Here we introduced an alternative method (SPF – the SVM-based Pathway Feature method) for discriminating enriched pathways by using SVM directly instead of GSEA to determine pathways which best discriminate the two classes of interest. Specifically, we built pathway features *P*_*j*_ using the training data in the same way as in section (2) above, with the exception that each aggregate feature *P*_*j*_ was constructed only from the top 20 genes (designated as *core genes*) in the corresponding pathway as selected using the Fisher method with separation criterion ${\left(\mu_{A}-\mu_{B}\right)}^{2}/(\sigma^{2}{}_{A}+\sigma^{2}{}_{B})$, representing the mean expression difference of a gene between the two classes, divided by the sum of the variances. We then used an SVM with features *P*_*j*_ in discriminating the classes in the training set, and obtained SVM weights *W*_*j*_ for each of these pathway features (obtained from combined data across the training samples *k*). Pathway rankings were now determined by the absolute scores |*W*_*j*_| in the training set. The top 20 pathways were selected (from the training set) for training and testing. In this case, we identified 20 core genes for each pathway with more than 20 genes; otherwise, we used all genes in the pathway. We chose 20 SVM feature selected genes per pathway since this number of selected genes approximately matched with the average number of GSEA- selected leading edge genes in the GLEG method (see above).

## Results and discussion

We analyzed and developed prognostic/diagnostic signatures for three different class distinctions: short term/long term survival and high stage/low stage for ovarian cancer, and metastatic/non metastatic tumor progression in breast cancer. We extracted gene expression profiles from The Cancer Genome Atlas (TCGA) for ovarian cancer phenotypes. These included 44 samples from 22 short term survivors and 22 long term survivors (SUR), and 59 TCGA samples differing in stage, with 10 early stage and 49 late stage samples. The ovarian survival and stage study subjects each individually yielded two separate sets of cancer tissue samples, whose biomarkers were extracted independently by groups at the Broad Institute (BI) and at the University of North Carolina (UNC). In addition, we separately analyzed an additional data set of gene expression biomarkers from 119 samples taken in a Duke University study [2] of advanced ovarian cancers. The phenotypes of the latter dataset were separated based on differential resistance to platinum therapy, with the data set separated into 34 incomplete response and 85 complete response samples. Finally, in order to test for biomarker stability across different types of data sets, we identified biomarkers that distinguished metastatic potential in breast cancer data from Wang *et al. *[3] and van de Vijver *et al. *[6] (details in the material and methods section).

We note that the UNC and BI ovarian cancer data sets in TCGA are based on tissue samples from the same subjects, analyzed by different laboratories, while the Wang and van de Vijver breast cancer data sets are analyzed by different laboratories and came from different patients.

### Evaluation of biomarker sets based on KEGG and MSigDB pathways

**Figure 2:**
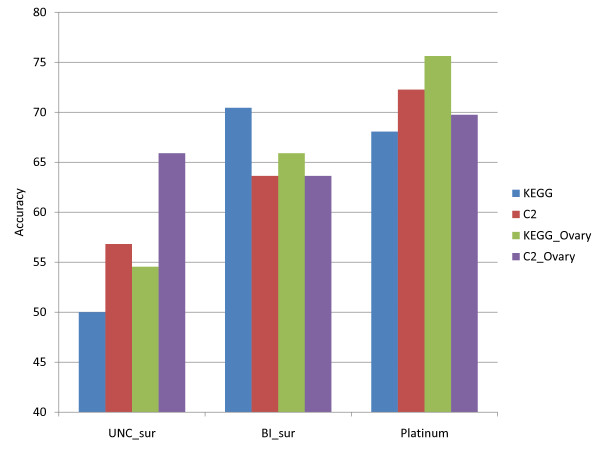
**Evaluation of the performances of different gene sets.** These implementations include 200 KEGG pathways (KEGG), 522 functional pathways (C2), 200 KEGG pathways with 5 curated ovarian cancer associated gene sets (KEGG_Ovary), and 522 functional pathways with 5 curated ovarian cancer-associated gene sets (C2_Ovary). All test data sets are extracted from primary ovarian cancer tissue. UNC_sur and BI_sur denote ovarian survival data sets analyzed from UNC and BI respectively, and Platinum denotes Platinum response data sets.

We first tested biomarkers based on the 200 KEGG [30] human pathways obtained from MSigDB version 2.5 [16] and compared them to an alternative set of aggregate biomarkers consisting of 522 functional gene sets (selected on the basis of pathway membership and other biological criteria) from MSigDB version 2.5 (data set C2 [16]). We remark that both the KEGG pathways and C2 functional gene sets in fact represented very limited total numbers of unique genes. Specifically, only 4128 and 5602 genes are covered by KEGG pathways and the C2 functional gene sets respectively. To accommodate the loss of gene information compared to the traditional method of starting with expression data for 12042 (BI) and 17814 (UNC) genes, we added 5 manually curated gene sets extracted from the literature, all associated with ovarian cancer [1,2,27-29], to the relevant KEGG pathway set (the expanded set is called KEGG_ovary) and to the C2 functional gene set (C2_ovary). Figure 2 gives an assessment of relative accuracies of classification using different functional gene sets using a support vector machine (SVM).

We remark that these accuracies are based on balanced data sets and pathway biomarkers which are created from leading edge genes from the most discriminative pathways. It is difficult from the data in Figure 2 to determine that one of the above four groups of functional gene sets is better than the others at discriminating classes.

### Evaluation of pathway-based classification methods based on ovarian cancer phenotypes

To evaluate pathway-based classification methods, we tested predictive performances for the different methods using pathway-based markers, as described in the Methods section. (a) The first is the GSEA-based Leading Edge Gene feature method (denoted here as GLEG). (b) The second is denoted as the GSEA Pathway Feature (GPF) Method. (c) The third is the SVM-based pathway feature (SPF) method.

In addition, in order to form a baseline measure, we created random gene sets as surrogate pathways by keeping KEGG pathway designations but doing a full permutation all genes (i.e. replacing each gene in a pathway by a randomly selected gene). We then performed the GPF pathway-based algorithm based on this randomly permuted gene set. The classifier based on this procedure was designated as the random pathway feature (RPF) method. We also calculated an additional baseline by selecting identical numbers of genes as the GLEG method from (i) the set of all KEGG genes, designated as the SKG (single KEGG gene) method and (ii) all genes (designated as SG).

All methods were implemented using standard SVM leave one out cross-validation in balanced data sets. Thus in the case of ovarian cancer stage classification, we randomly undersampled by choosing 10 samples out of 49 stage IV samples to balance 10 early stage samples, and repeated this procedure 10 times.

To compare with the baseline random pathway aggregation method, the accuracy of distinction between early stage and stage IV ovarian cancer using the UNC data set was 78% and 71%, respectively, using the GLEG and GPF methods. In comparison, it was 60% using the same number of randomly selected pathway features (RPF) as the number of pathway features in GPF. For the parallel BI stage data, the corresponding figures are 74% (GLEG), 81% (GPF) and 56.67% (RPF) respectively.

It can be seen from the results for the (random) RPF method (accuracies at 60% or less, uniformly lower than the GPF method), that the prior biological information from the pathways was a significant component of the method. In general, we have seen in other contexts that random clustering of features (which is the effect of this method of aggregating gene features into random pathways) can sometimes (surprisingly) improve performance over unclustered individual (single gene) features. In this case the random clusters (random pathways) did not outperform the individual features (genes), though even these RPF-based randomly clustered data conveyed some information.

**Figure 3:**
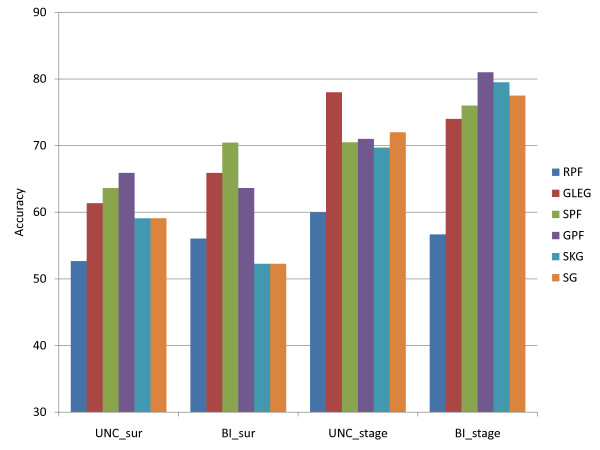
**Comparison of the performances of the RPF, GLEG, GPF, SPF, SKG, and SG methods.** These methods are tested in ovarian cancer data sets to discriminate survival time (SUR) and stage (stage). The notation represents use of the following features: RPF, random pathway features; GLEG, leading edge genes using GSEA; GPF, pathway features selected using GSEA; SPF, pathway features selected using SVM; SKG, single KEGG genes; and SG, single genes.

Figure 3 shows the remaining performances of the three pathway-based classification methods and the gene-based classification method for stage and survival in two different BI and UNC (TCGA) data sets.

### Comparative discriminatory accuracy of core gene and pathway markers within breast cancer data sets

**Figure 4:**
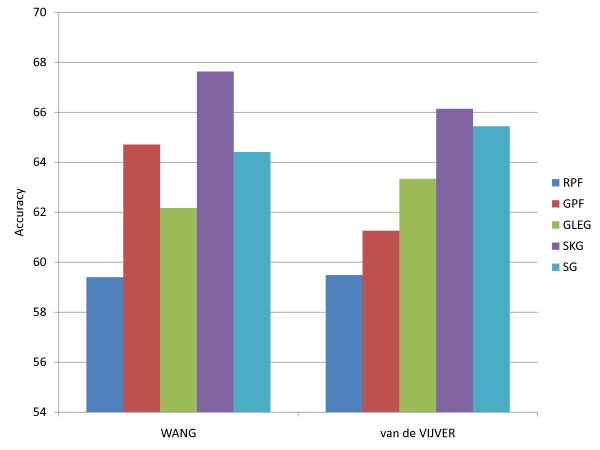
**Comparison of metastasis prediction performances based on the Wang and van de Vijver data sets.** Each data set tested 10 combinations of data subsamplings (for the purpose of balancing the data) with leave one out cross validation in each of the 10. The vertical axis shows the average accuracy. Here the RPF, GLEG, GPF, SKG, and SG methods were used.

To further assess the discriminatory accuracy of pathway biomarkers, we analyzed two large metastasis breast cancer data sets [3,6], both obtained from primary breast cancer, but from non-overlapping populations. Specifically, 93 patients in the Wang data set and 79 patients in the van de Vijver set were diagnosed with metastases within 5 years of initial diagnosis (metastasis group). The remaining groups of 183 and 216 patients, respectively, were designated as non-metastatic by the authors. For the Wang data set, we implemented 10 randomly selected subsampled data sets balanced (at *n* = 79 each) between metastatic and non-metastatic samples. These random sub-samplings of the Wang data set were performed in order to balance it between metastatic and non-metastatic cases. For each run on the balanced sets, leave-one-out cross-validation was performed. To compare discriminatory accuracy of pathway biomarkers against individual gene biomarkers, for each training data set (i.e., a new training set with each sample that was left out) we selected the top 20 upregulated and top 20 downregulated pathways separating the metastatic and non-metastatic groups, using GSEA. We then used the union of the leading edge genes from these 40 pathways as individual features. The classifier built on a training set was then used on the left-out test sample. The results are summarized in Figure 4. The best performing method was the SKG method, which uses the same number of individual gene features (selected only from KEGG) as the number of leading edge genes in the GLEG method. The obtained accuracies using this method are 66.94% (Wang) and 65.74% (van de Vijver). In contrast, using same numbers of genes not restricted to KEGG genes (SG) gives separate accuracies of 64.41% (Wang) and 65.44% (van de Vijver). The GLEG method gives values of 62.17% (Wang) and 63.34% (van de Vijver). The accuracies of the GSEA pathway feature method (GPF) for the van de Vijver and Wang data set are 61.26%, and 64.71% respectively.

To understand these results better, we will also briefly mention a two-fold cross validation we performed using the same methods on these two data sets. In this test we additionally performed a pathway-level feature aggregation using an averaging of pathway-level features, as opposed to the GPF method of combining gene features in pathways using their SVM weights. More specifically, we note that the pathway features obtained using the GPF method involve not only the leading edge gene expressions for a given pathway *p*, but that these expressions are weighted by the gene weights *w*_*pi*_ of these leading edge genes, inherited from the same training set when trained on the set of *all* genes. Depending on the noisiness of the data set, these weights may be unreliable individually. In particular, we noted that the weights in the van de Vijver data set were more unreliable than those in the Wang set. In fact, in this two-fold cross-validation test, accuracy in the van de Vijver data set was increased to 61.52% when we used *mean pathway features* generated without weights (still using leading edge genes only).

Briefly, we describe here an analysis of how such noise might have affected these additional two-fold results; however further research needs to be done on this topic. Assume that we separate gene expression levels into signal and noise components, i.e.,

(2) $${\tilde{x}}_{\mathrm{ij}}=x_{\mathrm{ij}}+z_{\mathrm{ij}}\text{,}$$

where *x*_*ij*_ is the gene expression of gene *i* in sample *j* (signal), and *z*_*ij*_ is the corresponding noise.

When we average gene expressions ${\tilde{x}}_{\mathrm{ij}}$ over a coherent subset of genes (e.g. when all genes are in the same pathway) the averaged noise *z*_*ij*_ is quenched, which can help reduce the signal to noise ratio. In the case where we use weights to obtain such pathway features, e.g., as in the weighted sum $\sum_{i\in \left\{\text{leading edge genes}\right\}}w_{i}{\tilde{x}}_{\mathrm{ij}}$, the weights will have additional error attached to them if they are obtained in a noisy training set. In this case the effect of replacing the above pathway feature with the averaged pathway feature $\sum_{i\in \left\{\text{leading edge genes}\right\}}{\tilde{x}}_{\mathrm{ij}}$ (with appropriate final normalization) allows avoidance of the noise inherent in overly noisy weights *w*_*i*_, as well as denoising by equal averaging of the test data noise terms *z*_*ij*_. However, if the noise in the weights *w*_*i*_ is qualitatively small enough, the performance of the weighted feature method can then improve on that of the above mean feature method. This phenomenon was observed when the same pair of methods (weighted and unweighted pathway averaging) was used on the Wang data set, and the weighted method performed better in this case than the unweighted one. This observation correlated with the fact that the weights in the van de Vijver data set had a significantly larger standard deviation than those in the Wang data set when different sub-samplings of the data were taken, leading to the conclusion that the van de Vijver data set was noisier. In this regard, combining these two methods (weighted and unweighted gene combinations) could be the most effective way to get better performance in this type of machine learning.

In the full leave one out experiment above, we also observed that all-gene features restricted to be obtained from genes in KEGG pathways (4128 of them) were more informative than features drawn from the full set of genes (over 12,000 in both data sets) available in the expression profiles.

### Accuracy of pathway biomarkers across data sets: Metastasis and ovarian survival data sets

To validate the stability of pathway-based biomarkers as well as their classification accuracy, we studied the expression profiles of the two cohorts of breast cancer patients [3,6]. In this study we used pathway features selected in one data set to predict metastasis in the other, thus effectively using one set as a training set and the other as a test set.

To determine pathway-based biomarkers, we determined in one data set the distinguishing KEGG pathways between the two phenotypes (metastatic and non-metastatic) using GSEA, and used these as biomarkers for classification of the other - we will call this reciprocal classification.

We tested each of the above pathway-based classification methods in this procedure, including the leading edge gene (GLEG) method and the pathway based biomarker (GPF) method. We compared these to standard (Fisher selection) SVM methods using matched numbers of genes as used in the GLEG method (SG method, see above). Out of 810 leading edge genes determined from the Wang data set, 636 of these were available in the van de Vijver data set. Correspondingly, there were 375 out of 391 unique genes chosen from the van de Vijver data set for reciprocal inclusion in the Wang data. The reason for the large difference in sizes is that numbers of leading edge genes were significantly different between the two sets. Thus the respective numbers of features using the GLEG method in the two data sets were 636 and 375.

**Figure 5:**
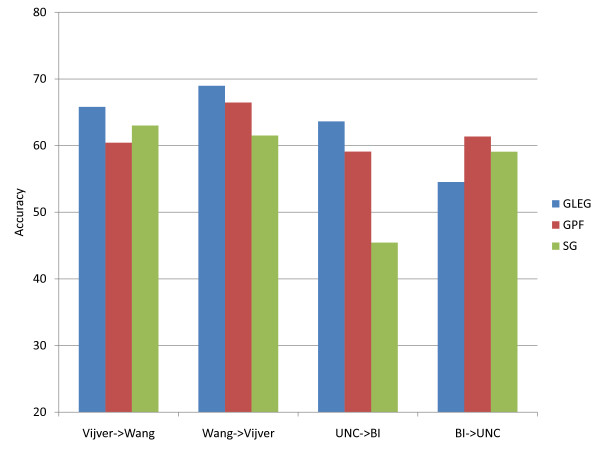
**Cross-validation between two different cohorts.** The accuracies of cross-validation between the Wang and van de Vijver data sets and between the UNC and BI survival time data sets. The arrow denotes that genes were selected from training data sets of one cohort and tested the genes in the other cohort. For example, Wang -- > Vijver means that genes were selected from the Wang data sets and tested in the van de Vijver data set.

Since both data sets are strongly unbalanced between two (metastatic and non-metastatic) phenotypes, we balanced the two classes for classification purposes (in training and test sets) by bootstrapping from the larger collection of non-metastatic samples using 5-fold undersampling, with each sample matched in size to that of the metastatic group. The performance figures (see Figure 5) form an average of 5 individual performances for each method on each data set. Figure 5 compares these reciprocal feature selection accuracies for pathway-based markers versus single gene markers.

For the reciprocal test, the leading edge gene (GLEG) method trained on the Wang data set achieved 68.97% accuracy in classifying metastasis in van de Vijver *et al. *[6], while the reciprocal accuracy (training using van de Vijver and testing on Wang data) yielded 65.83% accuracy (as before these accuracies are reported on data sets balanced between the two phenotypes). However, in this reciprocal testing the GPF method (using pathway features) performed better than the single gene (SG) method in testing on the van de Vijver data set and worse than the SG method on the Wang data set (Figure 5).

In the case of the ovarian survival time data, since the BI and UNC samples were obtained from the same patients, we divided each of these data sets into two groups (BI_group1, BI_group2, UNC_group1 and UNC_group2). The group 1 patients in the BI and UNC datasets were the same, and similarly for the group 2 patients. To maintain full independence (in both subjects and assay facilities) of training and test sets, we performed gene feature selection using BI_group1 to test UNC_group 2, and vice-versa.

**Figure 6:**
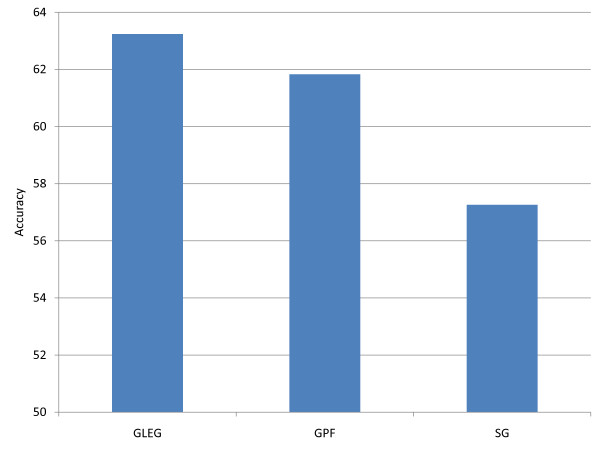
**Average of accuracies with respect to different methods.** The average of accuracies using the various methods for cross-validation between different data sets, such as the Wang and van de Vijver sets and the UNC and BI sets. The vertical axis represents averaged accuracies over all heights in the previous graph (Figure 5).

Figure 6 shows the average performance of each method. Overall, the best-performing method was the GLEG method, using leading edge genes based on GSEA pathway selection. The GLEG and GPF methods achieved average accuracies of 63.24% and 61.83%, respectively, among the four above-mentioned test data sets. Meanwhile, using single gene classifiers achieved average 57.26% accuracy. This is evidence that pathway-based biomarkers are more reliable for classifying cancer subtypes than single gene markers, in addition to their being more stable.

### Reproducibility of pathway-based biomarkers and single-gene markers between data sets

In order to test the robustness (i.e., stability across data sets) of the pathway-based biomarkers, we consider the ovarian cancer survival and stage data sets, and the metastatic breast cancer data sets mentioned earlier. We recall that the full UNC and BI data sets (used for the stage and survival analysis) are based on different ovarian cancer tissue samples from the same subjects (analyzed by different laboratories) and that the Wang and van de Vijver metastatic breast cancer data sets involved independent sets of patients and were analyzed by different laboratories. The primary purpose of our analysis here is to compare the stability of pathway/gene biomarkers between the following approaches: (1) use of Fisher-selected individual gene biomarkers as basic features (SG classifier); (2) use of pathway-selected leading edge gene markers (GLEG classifier); (3) use of enriched pathway biomarkers as obtained from GSEA (GPF classifier). Reproducibility was computed by dividing numbers of significant biomarkers (a) intersecting between two different experiments and (b) appearing in the union of those in the same experiments.

Specifically, if *B*_1_ and *B*_2_ represent the respective sets of significant biomarkers in the two experiments, the computed ratio is $=\left|B_{1}\cap^{\;}B_{2}\right|/\left|B_{1}\cup^{\;}B_{2}\right|$ , where |*A*| denotes the size of a set *A*.

In order to provide a valid comparison of the methods, we note that a comparison between an intersection and a union of two sets *B*_1_ and *B*_2_ as a measure of their generic mutual enrichment depends on the background (with total cardinality |*B*|) and the proportion of the background included in each of the sets, i.e.,|*B*_1_|/|*B*| and |*B*_2_|/|*B*|. In order to keep the above proportions of the background constant, we maintained all of them at 40/200, i.e., .2, which was the proportion of KEGG pathways we selected for the GPF method. Thus in comparing the stability of the GPF method with that of the SG (all single gene) and SKG (single KEGG gene) methods, we also selected from the above classes of genes the top 20% of all genes based on Fisher score, and formed a ratio parallel to the above ratio *S* for these single gene methods.

The reproducibilities *S* of pathway markers (based on the GPF method, i.e., proportions of top pathways in common among different data sets) are 0.40, 0.33, and 0.18 in the stage, survival, and metastasis data sets, respectively. The comparable figures for leading edge gene (GLEG) markers are 0.27, 0.25, and 0.15 (see discussion below). In contrast, the reproducibility of all single gene (SG) markers using Fisher selection are 0.22 in stage, 0.21 in survival and 0.07 in metastasis.

**Figure 7:**
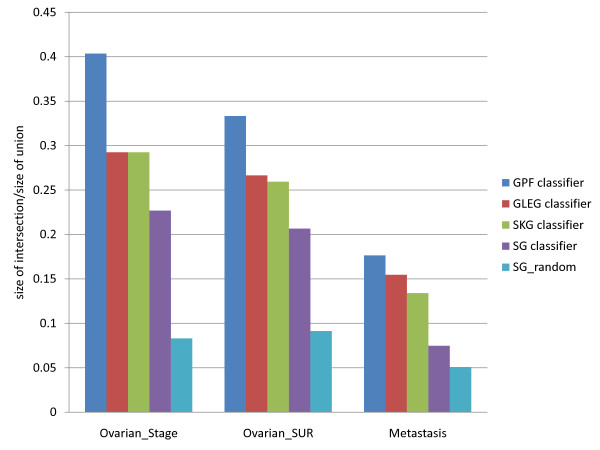
**Agreements of different types of significant markers.** The agreements of three different types of significance markers between two data sets: ‘GPF classifier’ denotes pathway features obtained by GSEA, and ‘GLEG classifier’ denotes leading edge gene markers determined by GSEA. ‘SG classifier’ and ‘SKG classifier’ denote the genes determined by Fisher selection from full gene expression profiles and restricted to the set of KEGG pathway genes, respectively, with feature numbers controlled to 20% of each population. ‘SG_random’ denotes gene sets selected randomly (again to 20% of the full gene set) from full gene expression profiles. ‘Ovarian_Stage’ denotes the ovarian stage data sets (marker stability compared between BI and UNC data), ‘Ovarian_SUR’ denotes ovarian survival data sets (BI vs. UNC), and ‘Metastasis’ denotes metastatic breast cancer data sets (based on the Wang and van de Vijver data sets). For each pair of datasets, overlapping biomarkers were all extracted from matching based on the top 40 pathways in each. Vertical axis represents biomarker consistency as the quotient formed by the size of the intersection of the two biomarker sets, divided by the size of their union.

These data are graphed in Figure 7. As shown there, the pathway/gene markers corresponding to a pathway-based analysis are more consistent than individual Fisher-selected gene markers selected directly from expression profiles.

We mention here that the above stability figures for the GLEG method (.27, .25, and .15) are generally underestimates of performance, since the numbers of leading edge genes which were generated by the top 20/20 (up- and down-regulated) pathways in fact amounted to be on the average 17% of all KEGG genes, yielding a smaller percentage than 20% of the background for the two sets *B*_1_ and *B*_2_ mentioned above. Therefore, as discussed above, since a larger percentage of the background can only improve the consistency ratio *S* defined above, this is in fact a slight underestimate of the performance of the GLEG method.

**Figure 8:**
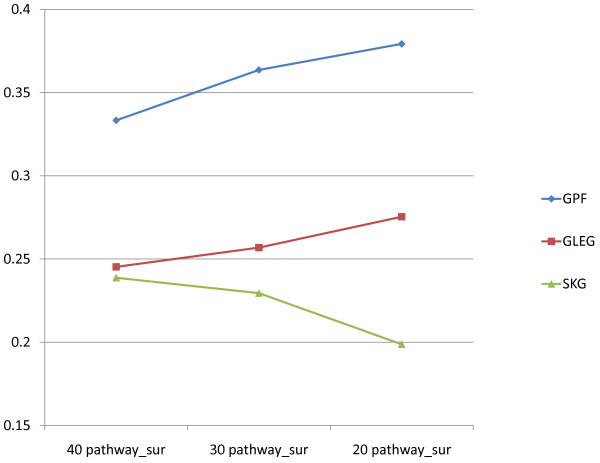
**Consistency of different classes of biomarkers with respect to numbers of candidate pathways.** The consistency (overlap level) of different types of top gene/pathway markers based on varying numbers of selected candidate pathways (40, 30, or 20) between UNC and BI ovarian survival data sets. For example, the 30 pathway_sur column heights represent overlap percentages of biomarkers in the survival data sets from BI and UNC (using pathway, leading edge gene, and single gene biomarkers, respectively, all extracted from matching based on the top 30 pathways in the BI and UNC datasets). Vertical axis is defined as in Figure 7.

The above results indicate the overlap based on top pathway markers consisting of 20 up-regulated and 20 down-regulated pathways in each data set. Since we considered the BI/UNC pathway overlap to be less noisy, we also attempted a more parsimonious test of overlap between the two datasets using only 10/10 (upregulated/downregulated) pathways in each dataset. A significant pathway overlap signal was obtained also in this case. The result for different pathway numbers among these data sets is given in Figure 8. In particular, the highest signal *S* in pathway markers is .38 at a level of 10/10 (up and down-regulated) for survival data.

The proportion of common pathways between the UNC and BI data sets increased from counts with 20/20 pathway selections to 10/10 selections, and the same holds for the selected leading edge gene ratios. In contrast, the ratio for Fisher-selected genes decreased at the same time. This implies that top pathways and pathway-based genes (leading edge genes) contained more core gene sets/pathways as stable biomarkers. The identical pattern of overlap for pathway markers, leading edge gene markers, and Fisher selected markers was observed for stage data.

Lower pathway overlap numbers for the 10/10 case in the Wang/van de Vijver data sets gave a clearly less significant signal (an overlap of only 3 pathways), presumably because of the higher variability involving both subjects and measurement protocols.

We remark that the performances indicated in Figure 8 may have the following interpretation. The decline in performance of the SKG (single KEGG gene) method as the number of pathways decreases from 40 to 20 may result from the following fact. First, for relatively small sample sizes (there were 22 short and 22 long survival cases), the Fisher method of differential expression measurement is not robust, so that as the number of potentially matching genes in the two sets decreases, the SKG curve of Figure 8 indicates a corresponding decrease in overlap of these genes.

We note that the overlap in enriched pathways vs. overlap in individual genes between the UNC and BI tissue samples is a measure of stability against variance in different measurements from the same individuals, as opposed to bias introduced by comparison of samples from completely different individuals. In contrast, the pathway stability vs. gene stability studied in the analysis of breast cancer metastasis (among the Wang and van de Vijver data sets) is a measure of stability against both the bias of an entirely different population as well as the variance of different sets of measurements.

### Informative genes based on leading edge and Fisher-selection markers

In determining biologically significant biomarkers for differentiating two phenotypes (e.g. metastatic vs. non-metastatic cancer), it is generally more powerful to find significant biomarkers overlapping in biomarker selections from several different methods. Here we have selected significant markers based on the pathway method (based on leading edge genes, Table 1), discussed above, in addition to then refining these by also using the standard Fisher single gene selection method (Tables 2 and 3 below).

**Table 1:** Invariant genes (between datasets) among common leading edge ranked by average p-value

**Breast gene sets**	Average p-val *1.0e-04	**Ovarian gene sets**	**Average p-val**
CCNE2	.0029	ID4	.0011
MAD2L1	.0910	DPYSL3	.0049
SQLE	.1071	FBXL7	.0132
CCNB2	.1103	POLR1D	.0134
PSMA7	.1140	EDAR	.0138
HLA-DRA	.2229	ANXA4	.0185
CCNA2	.2591	CXCL9	.0198
E2F1	.3749	BMPR2	.0204
BUB1B	.4985	MYLK	.0216
TSTA3	.5691	ALDH5A1	.0241

**Table 2:** Invariant genes (between datasets) among common leading edge and Fisher-selected genes: Wang and van de Vijver data sets - see Informative Genes section for summary citations below

**Gene symbol**	**Full name**	**Summary**
MAD2L1	Mitotic arrest-deficient2, S. Cerevisiae, homolog-like 1	Overexpressed in breast and ovarian cancers [31,32]
PTTG1	Pituitary tumor-transforming gene 1	Oncogene related with breast cancer [33]
BUB1B	Budding uninhibited by benzimidazoles1, S. Cerevisiae homolog of beta	Overexpressed in breast cancer [31]
SQLE	Squalene epoxidase	Overexpressed in breast cancer and significantly inversely related to distant metastasis-free survival in early stage breast cancer [34]
E2F1	E2F transcription factor 1	Related with breast cancer outcome [35]
TSTA3	Tissue specific transplantation antigen P35B	Conserved in several breast cancer subtypes [36]

For each dataset common Fisher and leading edge genes were selected, and then intersected between the two datasets. Genes previously connected to cancer (with summaries) are listed here, and remaining genes are included in (Additional file 1: Table S1).

**Table 3:** Invariant genes (between datasets) among common leading edge and Fisher-selected genes: UNC and BI data sets – see Informative Genes section for summary citations below

**Gene symbol**	**Full name**	**Summary**
ID4	Inhibitor of DNA binding 4	Inhibitor of BRCA1 in breast and ovarian cancer [37]
ANXA4	Annexin A4	Identified as related with chemotherapy-resistant clear cell ovarian tumors [38]
CXCL9	Chemoline (C-X-C motif) ligand 9	Breast cancer-related gene [39]
MYLK	Myosin light chain kinase	Related with breast cancer [40]
FBXL7	F-box and leucine-rich repeat protein 7	Breast cancer [41]

See above caption (Table 2); remaining genes are included in (Additional file 1: Table S1).

We first present the 10 most significant common leading edge genes from the two metastasis data sets, and then those from the two ovarian survival data sets, in Table 1. These genes were obtained by intersecting the leading edge genes in the top 20/20 pathways (20 upregulated and 20 downregulated) between the Wang and van de Vijver data sets. This resulted in 161 genes in common. The 10 genes with the highest averaged *p*-values (between the two datasets) were then selected (Table 1).

In addition to this method for identifying stable discriminative genes, we combined the pathway-based marker selection method with the standard Fisher *p*-value method (Tables 2 and 3) as follows. Among the Wang and van de Vijver metastasis data, we first identified a total of 118 genes and 70 genes, respectively, representing the intersection of the leading edge genes and a matched number of top Fisher selected genes, obtained separately in the two studies. These genes represented significantly up- or down-regulated genes between metastatic and non-metastatic patients. Between these two sets (118 genes from the Wang and 70 from the van de Vijver data), a total of 13 genes overlapped (see Additional file 1: Table S1). This is significant in that the original overlap between the two sets of top genes differentiating metastasis (also using the Wang and van de Vijver data sets), numbering 76 and 70, respectively, consisted of only three genes [5]. We note that 9 out of 10 genes in Table 1 are also found among the 13 genes in Additional file 1: Table S1.

The above 13 genes from the metastasis data were CCNB2, PSMA7, CCNE2, PTTG1, TPI1, RRM2, MAD2L1, BUB1B, SQLE, E2F1, NP, PSMB5, and TSTA3. Among these genes, 8 (consisting of PSMA7, TPI1, SQLE, E2F1, PTTG1, TSTA3, BUB1B, MAD2L1) have been confirmed in the literature [31,33-36,42] to be involved with several different cancers. Additional file 1: Table S1 presents the full name, designation and annotation of each gene. In addition, among the above 8 genes, SQLE, E2F1, PTTG1, TSTA3, BUB1B and MAD2L1, have been related with breast cancer [31,33-36]. In particular, SQLE has been confirmed to be a predictor in early stage breast cancer of freedom from distant metastasis. Recently the pituitary tumor transforming gene PTTG1 was reported as an oncogene associated with breast cancer [33] as well as regulation of the immune system [43]. Vuaroqueaux *et al. *[35] reported E2F1, a well-known key transcription factor in proliferation and apoptosis, as a surrogate marker of breast cancer outcome. TSTA3 has been found to be one of the conserved genes for several breast cancer subtypes such as luminal, ERBB2+, and basal [36]. Yuan *et al. *[31] showed that MAD2L1 and BUBlB, known as spindle damage checkpoint genes, were overexpressed in breast cancer tissues.

Among the UNC and BI survival data, 11 genes, consisting of POLR1D, ID4, EDAR, BMPR2, HLA-DOA, DPYSL3, ANXA4, CXCL9, MYLK (MLCK), FBXL7 and TBL1X, overlapped among both the leading edge and Fisher genes in both datasets (forming four collections of genes among them). Among these 9 of the genes, consisting of POLR1D, ID4, HLA-DOA, DPYSL3, ANXA4 CXCL9, MYCK (MLCK), FBXL7, TBL1X are directly or indirectly related with cancers such as breast, endometrial, brain, colon, ovarian, and B-cell cancers [37,39,40,44-48]. Additional file: 1 Table S1 shows the full name of each gene and the related cancer. In particular ID4 was confirmed an inhibitor of BRCA1 in ovarian and breast cancer by Welcsh and King [37]. ANXA4 has been proposed to be related to chemotherapy-resistant clear cell ovarian tumors by Kim *et al. *[38], and also found in clear cells of ovarian and endometrial cancer by Zorn *et al. *[44]. Table 2 shows the information on top genes which are strongly related with breast cancer and Table 3 shows the same for ovarian cancer.

In addition, 6 genes consisting of CCNB2, CCNE2, PTTG1, MAD2L1 BUB1B, E2F1, are all found in the cell cycle pathway, which was found to be enriched in metastatic tissues (see next section). In the case of ovarian cancer, EDAR and CXCL9 are found in the cytokine-cytokine receptor interaction pathway, while ID4 and BMPR2 are found in the TGF signaling pathway, and TBL1X is found in the Wnt signaling pathway. In addition, four of the ovarian genes (DPYSL3, ANXA4, MYLK, and FBXL7) were in ovarian cancer module [28], one of our 5 curated sets of ovarian cancer-related genes. All pathway information for each gene is provided in Additional file 1: Table S1.

### Enriched pathways in ovarian survival and breast cancer metastasis data

We began with the top 20 discovered common ovarian cancer pathways between the BI and UNC data sets, forming the intersection of the top 40 in each (originally selected as half up-regulated and half down-regulated). These pathways were selected from the collection of all KEGG pathways, together with the above-mentioned 5 ovarian-related gene sets which we had curated independently of these data (see Methods). We also obtained the 12 common pathways (again out of 40 each) from the two breast cancer metastasis cohorts, this time selected strictly from KEGG pathways. A number of these common pathways (both from the ovarian and breast cancer datasets) have had independent verification as being cancer-related (Additional file 2: Table S2), primarily in the context of differentiating cancer and normal tissue. Based on their validation, the significance in our differentiating cancer phenotypes (survival and metastasis) is also of interest.

We now mention some previously studied cancer-related common enriched pathways, whose functions are described in Tables 4, 5 and 6. Three pathways, consisting of type 1 diabetes mellitus, cytokine-cytokine receptor interaction and hedgehog signaling, are in common between the ovarian long survival and breast cancer non-metastasis groups. In particular, 8 out of 9 common leading edge genes common to both the ovarian and breast cancer data sets in the type 1 diabetes pathway are in the HLA family of immune system activators.

**Table 4:** **Selected common enriched pathways: breast cancer and ovarian cancer (all enriched in long survival/non-metastasis)** Enriched pathways in both ovarian and breast cancers

	**Function of pathway**
HSA04940_TYPE_1_DIABETES_MELLITUS	Includes human leukocyte antigen (HLA) gene family, related with immune system function for protection against cancer and mediation of autoimmune disease.
HSA04060_CYTOKINE_CYTOKINE_RECEPTOR_INTERACTION	Cytokines can control invasion and metastasis, and also function to inhibit tumor progression [49]
HSA04340_HEDGEHOG_SIGNALING_PATHWAY	Crucial role in tumorigenesis and cancer growth and spread [50]

This table contains pathways (with known relations to cancer) common to enrichment for ovarian survival and breast cancer non-metastasis. Tables 5 and 6 list cancer-related pathways enriched individually for these two phenotypes.

Enriched pathways in both ovarian and breast cancers.

**Table 5:** Selected common enriched pathways: BI and UNC ovarian data (all enriched in long ovarian cancer survival)

**Common long survival pathways between BI and UNC**	**Function of pathway**
HSA04514_CELL_ADHESION_MOLECULES	Known to interfere with cellular detachment and cancer metastasis, related with cancer invasion and metastasis [51]
HSA04310_WNT_SIGNALING_PATHWAY	Includes oncogene and tumor suppressor genes [52]
HSA04612_ANTIGEN_PROCESSING_AND_PRESENTATION	Biological process for preparing antigens for presentation to special cells of the immune system.
HSA04350_TGF_BETA_SIGNALING_PATHWAY	Effecting on tumorigenesis either negatively or positively [53]

**Table 6:** Selected common pathways between van de Vijver and Wang datasets (all enriched for breast cancer non-metastasis; bold for those enriched for metastasis)

**Common pathways for non-metastasis (or metastasis in bold)**	**Function of pathway**
HSA04610_COMPLEMENT_AND_COAGULATION_CASCADES	Coagulation inhibits lung cancer metastasis in animal studies [54] A part of innate immune system and an effector of antibody-mediated immunity [55]
**HSA00100_BIOSYNTHESIS_OF_STEROIDS**	Found in metastatic tissue [56] and related with feminizing syndromes [57]
**HSA04110_CELL_CYCLE**	Related with cell growth and cell death

The set of common (breast and ovarian) leading edge genes in the cytokine- cytokine receptor interaction pathway (upregulated in survival/non-metastasis) consists of four genes, BMPR2, KIT, TNFRST11B, and IL1B, the last three of which are known immune system-related genes. The leading edge genes in this pathway differentiating only the ovarian survival datasets include five members of the chemokine ligand (CXCL) family, including one chemokine receptor, as well as four interleukin (IL) members coding proteins embedded in the cell membrane of immune system cells, including T and natural killer (NK) cells. In the breast cancer metastasis data, the cytokine pathway leading edge genes included eight IL members and 5 tumor necrosis factor receptor superfamily (TNFRS) members which activate immune system cells. The hedgehog signaling pathway is associated with ovarian cancer in that its deregulation is frequently observed in epithelial ovarian tumors [50,58], though this upregulation is not observed in all cases [59]. It has also been observed to be upregulated in breast cancer [60]. Nevertheless, this pathway’s upregulation in both the breast metastasis/ovarian survival data strongly indicates that the proper normal functioning of the pathway as a growth and development regulator may also be important in prevention of metastasis and growth and thus in patient survival.

The cell adhesion molecules, Wnt signaling, antigen processing and presentation, and TGF beta signaling pathways were enriched pathways in long survival for ovarian cancer. These pathways are consistent with interpretations as tumor suppressor and immune system pathways (see Tables 4 and 5). The Wnt signaling pathway has arms which both promote cell proliferation and apoptosis, and correspondingly is associated with both tumor promotion and tumor suppression [58,61,62], though in ovarian cancer its enrichment in long survival time indicates the latter role. In particular its upregulation in ovarian survival indicates a role which in its correlation with higher survival time contrasts with its upregulation in tumors vs. normal tissue. Its key role in ovarian cancer (and in the present cohort as an apparent growth regulator when it is functional) adds to known information on its noted dysregulation in a number of ovarian cancers [58,61,63]. Though the latter information is based primarily on comparisons of activations of Wnt in cancer vs. normal tissue, the analysis here differentiates cancer tissues from each other with regard to metastasis.

Among pathways enriched in the metastatic breast cancers, the cell cycle, and biosynthesis of steroids pathways have been observed as overexpressed in tumorigenesis in prior research (see Tables 4 and 6). In contrast, the complement and coagulation cascades, enriched in non-metastatic cancers, is known to protect against tumors by activating the immune system [55].

Additional file 2: Table S2 shows the common enriched KEGG/ovarian pathways for ovarian survival (between BI and UNC) and those for breast cancer metastasis (between Wang and van de Vijver). In the case of ovarian cancer, 7 out of the 20 pathways have been found significant in a previous study (Dressman *et al.*, note * in Additional file 2: Table S2).

We note that women who carry certain high levels of risk factors for breast cancer (e.g. family history) are at least 15 times more likely to develop ovarian cancer than non-carriers [64]. Thus, the three common enriched pathways between ovarian cancer survival and breast cancer non-metastasis present themselves as prospective candidates for further investigation on the relationship between the two diseases.

## Conclusions

Recently, human pathway information databases have been growing dramatically, enabling further opportunities for understanding molecular mechanisms of cancer and its subtypes, connectivity of diseases, mechanisms of drug action, etc. In addition, improving coverage of human pathway information has enabled more precise diagnosis of disease states, and consequently better supervision of patient treatments, including drug therapies. Currently, the integration of pathway information and gene expression profiles is becoming a useful tool for clinical practice. In this context, we are proposing a method for using pathway-based biomarkers to discriminate disease states for use in clinical prediction and treatment. The emphasis in this paper is stability of such biomarkers across data sets as a means to standardize analysis of biomarkers and consequently to help in potential clinical applications.

We have demonstrated that such pathway-based biomarkers (pathway features and leading edge gene markers) are significantly more consistent than single gene markers among different data sets. Additionally, the pathway-based markers have improved the classification accuracy of disease status both when classifiers are trained on different data sets and within cross-validated single data sets. Using genes selected by pathway-based programs such as GSEA has improved classification overall, though such improvement has not been universal. Herein, we need to undertake the task of exploiting these verifiably more stable biomarkers to find improved classification methods for disease status and prognosis.

The increased stability of biomarkers based on pathways has resulted in isolation of significant ovarian and breast cancer pathways which have been independently identified as being cancer-related. The three pathways (type 1 diabetes mellitus, cytokine-cytokine receptor interaction, and hedgehog signaling) which are common between breast and ovarian cancer have been previously identified as having particularly strong connections with cancer.

## Reviewers' comments

Reviewer 1: John F. McDonald (nominated by I. King Jordon)Reviewer comments:The authors used a Support Vector Machine (SVM) algorithm in order to develop three novel methods for discrimination of gene expression data sets and the identification cancer biomarkers. The established GSEA software application (Gene Set Enrichment Analysis) and the Fisher’s separation criterion were used in combination with SVM-weighted parameters. The three novel methods, GLEG (GSEA-Leading-Edge-Genes), GPF (GSEA enriched-Pathway-Features) and SPF (SVM-Pathway-Features) were designed for identification of gene-based and pathway-based biomarkers using microarray data. The authors argue that these methods are more accurate and reproducible across different data sets and different platforms compared to traditional methods.The authors tested the accuracy and the reproducibility of the methods across different platforms and across different data sets. They also compared their three novel methods with the standard gene-based biomarker methods (i.e. differentially expressed genes) and pathway-based biomarker methods (i.e. enriched pathways of differentially expressed genes). For all of their comparison tests they used two data sets. The first data set consisted of 287 samples from ovarian cancer patients with different survival times (short survival of less than 1 year vs. long survival of more than 1 year). The second data set had 286 breast cancer samples from one study and 295 from another. Both of the breast cancer sets had samples with different metastatic potential (i.e. samples that showed metastasis in 5 years after initial diagnosis vs. samples with no metastasis within the same timeframe). The ovarian samples were analyzed two times (from different research groups) using different platforms (Affymetrix and Agilent). The breast cancer samples were two separate studies, the first analyzed by Affymetrix U133A and the second by a platform fabricated by an ink-jet oligonucleotide synthesizer.The proposed novel methods are straightforward in design and implementation but they do not outperform the traditional methods in all comparisons (achieving accuracy of only ~60%). Regardless, one of the three methods (GLEG) appears to be reproducibly the best across the various data sets.The findings reported in this paper are noteworthy and worthy of publication. Nevertheless, there are issues with the paper that made it more difficult to read than is necessary. The following suggestions are offered to the authors in the spirit of possibly improving the presentation. I don’t consider these comments mandatory but I think the authors should consider them as suggested ways to possibly improve what is basically and sound and interesting paper.Abstract· Background:“2-level hierarchical feature vectors”: the 2-levels of the vectors might be briefly explained here.“The methodology extends…from RNA-Seq tools…”: the application to RNA-Seq is mentioned here but there is no example demonstrated in the entire study- therefore this comment should be omitted.[Authors’ response]We thank Prof. McDonald for making these points – we have expanded explanatory remarks regarding hierarchical feature vectors in the abstract and Background and have addressed his comments regarding RNA-Seq. Regarding the latter, see also the discussion below (Background section) on this.Reviewer comments:Background· The second paragraph mentioning the protein-protein interactions network example is not totally relevant to the gene expression analysis yet it is described in length. If it was to serve as an example, the protein-protein interaction study should not require more than two sentences for description.Also the last sentence of the same paragraph “Protein-protein interaction-based as well as coexpression-based aggregations have been shown in various contexts to improve performance of classification methods” needs reference.· The third paragraph could be made part of the sixth paragraph and both might be more appropriately placed at the end of the Background/Introduction section.· The fourth and fifth paragraph could be combined. The fifth paragraph is unnecessarily detailed and could be shortened in length or omitted.· In the sixth paragraph, in the third sentence: “This can provide…with feature vectors”: --“feature vectors” might be briefly explained.· The seventh paragraph could be omitted (there are no further examples on RNA-Seq in the paper so there is no need to be discussed at that point).· The tenth paragraph could be omitted or added to the Discussion section. Some notions like the benefits of the pathway-based biomarkers or the introduction of the multi-level aggregation approach might be more appropriate for the Discussion.· The fourteenth and fifteenth paragraphs might be omitted or re-written. They do not seem to flow coherently with the rest of the text. The authors might consider placing them towards the beginning of the section.[Authors’ response]The reviewer’s comments are reflected in the current composition of the paragraphs mentioned.Regarding RNA-Seq, the intent of this discussion is to indicate that with current changes in gene expression technology, the methods in this paper apply as well to gene-level expressions derived from RNA-Seq as to standard microarrays. Specifically, RNA-Seq data can be converted to more useful gene-level expression data whose form exactly parallels microarrays, assigning a single expression level value to each gene. These comments therefore point out that the methods of the paper are to this extent platform-independent. This is reflected in the current form of the discussion in the paragraph.Reviewer comments:Materials· The second half of the first paragraph “In order to…ovarian cancer.” and the second paragraph should be incorporated in the Methods section. The Materials section should describe (and not explain in detail the reasons of) the data utilized in the project.[Authors’ response]We feel that this description of the sub-selection of the dataset is so closely tied to the discussion of the ovarian cancer dataset itself that some meaning would be lost if this were moved. Therefore we would like to keep these sentences in their current location as a means of clarifying the use of the data.Reviewer comments:Methods· In the first paragraph, the sentence: “In general…(see diagram).” Is unclear. It is unclear what diagram is being discussed.· In the second paragraph, the phrase: “…SVM-based pathway selection method” needs a reference at the end (i.e. where the reader should refer to go and read for a detailed description of the method)· The following descriptions of 1), 2), and 3) methods need a general title (e.g. Novel Gene and Pathway based biomarker identification algorithms).· 1) The GLEG method:- In the first sentence the reference at the end is not numbered.- The explanation of how the GSEA p-values are calculated may be omitted (the reader may use a reference of the GSEA to find further details). In the last paragraph, the “training subset of the data” is not explained. Also, the “test set” is not explained either.· 2) The GPF method:- In last paragraph, the P_j is mentioned as “the pathway activation”: a pathway is activated if the downstream genes are expressed at sufficient levels to activate the downstream processes of the pathway. Since this is not the case for every enriched pathway of the algorithm, then better to mention “P_j is the relevant pathway” and not “P_j is the pathway activation”.[Authors’ response]We have integrated the intent of the reviewer’s first four comments regarding the Methods section of the paper. Since the three feature selection methods and their descriptions are integrated into the Methods section, we would prefer to maintain their numbered structure as a part of that section.Regarding the use of the term pathway activation for the term P_j, we are reflecting usage which has appeared in publications prior to ours – we have explained in the background section our (and other papers’) usage of this terminology.Reviewer comments:Results/Discussion· Evaluation of biomarker sets based on KEGG and MSigDB pathways- The human pathways in KEGG are currently 248. The authors used 200 but do not justify their selection of 200 out of the 248.· Evaluation of pathway-based classification methods based on ovarian cancer phenotypes- In the first paragraph it may not be necessary to explain the methods (a), (b) and (c) (i.e. the GLEG, GPF and SPF). These were already mentioned in detail in the Methods section. The last part of this paragraph “In addition, in order to form a baseline measure…(designated as SG)” that describes the randomized method, could be added in the Methods section.- In the second paragraph, the authors mention the accuracy of the randomized method, the GLEG, the GPF and the RPF, but they have not used the SPF method and they do not rationalize their decision.· Comparative discriminatory accuracy of core gene and pathway markers within breast cancer data sets- The second paragraph describes a two-fold cross validation- should this also be added to the Methods section?· Reproducibility of pathway-based biomarkers and single-gene markers between data sets- In the second paragraph the authors do not define what |B| represents. Even if obvious, every symbol of an equation needs to be defined.- In the sixth and seventh paragraph, the selection of 10 up regulated and 10 down regulated pathways instead of 20 up and 20 down, is a method not described in the Methods section.[Authors’ response]Regarding the version of KEGG used in the paper, we downloaded pathway information from MSigDB Version 2.5 [16]. This was the most current version when we began this work, which at that time listed 200 pathways. As to the description of the performance of the SPF method in this section, this is summarized in the graph in Figure 3, along with the performance of the other methods. Regarding the definition of |B| for a set B, this definition is already given in the “Reproducibility of pathway based biomarkers” section. The Results/Discussion section reflects the remaining comments of the reviewer.Regarding the choice of number of up and down-regulated pathways, this was determined by the need for an overlap size which was in a range where reasonable comparisons between the different methods could be made (i.e., out of 40 pathways on each side we would have a typical overlap of 15 or so). We varied this number in some cases (in particular in the ovarian datasets) so that we had a total of 20 pathways (10 up and 10 down) on each side, when sufficient overlap could be used to compare different methods. We feel that the need for this variation in pathway numbers was sufficiently described in the results section.Reviewer 2: Eugene KooninReviewer comments:This review provided no comments for publicationReviewer 3: Nathan J. Bowen (nominated by Dr I. King Jordan)Reviewer comments:In this manuscript, the authors introduce methodologies for integrating both gene level and pathway level gene expression differences for discriminating between cancer subtypes and clinical outcomes. I find the manuscript to be very well written, experimentally sound and the conclusions to be accurate.The authors use hierarchical SVM in order to enable the use of both genes and pathways in their discriminatory analyses.The methodologies introduced here take advantage of the notion that deregulated pathways, which contain many genes, are likely to emerge as more statistically significantly discriminators of cancer subtypes than individual gene sets by themselves. The incorporation of pathways into subtype and outcome prediction methods doesn't rely on the exact same genes being detected by microarray or RNA-seq - methods that still contain certain levels of stochasticity - but allows for the identification of an overlapping subset of genes from a pathway as being differentially expressed in order to increase discriminatory accuracy.In Figure 6 the authors summarize that the discriminatory accuracies of their pathway incorporating methods (GLEG and GPF) are higher than those for Fisher-selected individual gene biomarkers (SG). However the accuracies are improved from 57% for SG to ~62% and ~63% for GPF and GLEG, respectively.While I feel that the authors’ methods are sound, reproducible and use rigorous data and mathematical classification systems (actually far beyond my level of expertise), I am concerned that the improvement in accuracy is so small. This is not a reflection of the quality of the manuscript, just an observation/opinion of the results. It is unlikely that this small increase in accuracy will impact clinical decision making in the short-term future. However, as data sets grow larger, the incorporation of GSEA pathway and leading edge genes into classification schemes will likely increase the effectiveness and implementation of methods such as those presented here in clinical decision making.Currently, the use of terms such as "personalized medicine" are in vogue when interpreting molecular data and implementing clinical regimes. And there is data indicating that even tumors of the same organ and subtype are unique in many ways (e.g., somatic mutations).It may be that we are reaching the maximum level of discriminatory power for tumor subtype and clinical outcome by using molecular features that are in common among tumors.Do the authors feel that the accuracies of their methods will improve with more data?Or have we reached our limit of classification based on common genes and pathways?Other possible typo error-The Figure 6 legend refers to the previous figure as Figure 4, I think the authors are referring to Figure 5.[Authors’ response]We thank the reviewer for his comments.We agree with the reviewer that the accuracy improvements of this method, while positive in almost all cases, do not form a basis on their own for justification of its use. The key element of the use of this method is the principle of integration of biomarkers into coherent canonical combinations, i.e., combinations with a natural easily replicable form in different medical/biological contexts. In particular we feel a main accomplishment of this paper is a verification that such pathway markers are reproducible in significantly greater proportions than individual gene markers. For example, in the case of breast cancer metastasis prediction, the percentage overlap in pathway markers between the unrelated studies of Wang [3] and van de Vijver [6] is significantly higher than individual significant gene marker overlaps. This can be shown not to result from the smaller number of pathways, but rather because of the increase in their individual reproducibility. The discussion in the Reproducibility section of the results in the Discussion section addresses these questions (see also Figure 7).We expect that the use of such canonical markers as the P_j pathway markers will increase as the desire to standardize, recognize and understand individual biomarkers increases. We agree with the reviewer that standard classification methods which purely rely on a dataset without any external information have reached an asymptote in their discriminative ability. However, the knowledge in the structure of the pathway markers involves more information than just the dataset – it also involves domain knowledge in biology which identifies the gene groups forming pathways which act coherently enough to improve and standardize classification in the future.We thank the reviewer for these and his remaining observations.Reviewer 4: Ekaterina Kotelnikova (nominated by Mikhail Gelfand).Reviewer comments:The idea to use a pathway-based approach to biomarker discovery seems to be quite promising, since the intersection between suggested biomarker panels from different publications is often very poor, suggesting that standard data mining approaches are not sufficient in this case.However, the proposed approaches should be described and confirmed in more detail, i.e.1. There are no parameters for the SVM algorithm presented.2. There is no particular rationale for the number of top enriched pathways being used (just general words about non-trivial intersection without any numbers or test datasets).3. There is no rationale for the number k’s selection (number of discriminatory genes in the pathway). In addition to this information, it would be useful to discuss and substantiate the idea of the same k selection for different pathways. Since pathways are quite different in the number of their members, the same number k can lead to overfitting for one pathway, and could be insufficient for another.4. For all figures (and corresponding places in the text) related to the accuracy assessment the confidence intervals should be added. Without these numbers, the superiority of pathway-based approaches could not be claimed.5. For all figures (and corresponding places in the text) the comparison with random gene set-based approach with corresponding confidence intervals should be added (now this information is missing for several of them).As soon as these comments will be addressed, I think that the manuscript can be recommended for publication.[Authors’ response]We thank the reviewer for her observations, which we will address individually.1. We have incorporated the reviewer’s suggestion on parameters for the SVM.2. Regarding the discussion on the numbers of pathways chosen, we have also responded to a similar question from Reviewer 1. We selected the top 20 up-regulated and 20 down-regulated pathways so that we could optimally tune the sensitivity of the number of common pathways between two different experiments; this is also detailed in the paper.3. Regarding the question on the number of genes chosen in a pathway, we have clarified this discussion in the paper. The primary tool involves a feature selection method which is orthogonal to the classifier itself, and this is done in the GLEG method. We have added to our explanation that we wanted to compare this primary gene-level feature selection method with a baseline SVM feature selection approach, for which we chose 20 genes to reflect a figure close to the average number of leading edge genes chosen.4, 5. Because of small sample sizes we used leave-one-out cross-validation in order to obtain the largest training sets possible. With this procedure we essentially had only one data set available, making it impossible to produce error bars on the diagrams. We agree that this is a desirable element of such a study, and would like to pursue this possibility in a future study with larger data sizes. Randomization of pathway structures was done using the RPF method, and showed the significant role that pathway structures play in predictability of the cancer outcomes in ovarian and breast cancer. We appreciate the reviewer’s suggestion regarding further randomization, which would be a useful baseline comparison in later work.

## Abbreviations

GSEA: Gene set enrichment analysis; SVM: Support vector machine; TCGA: The Cancer Genome Atlas; GLEG: GSEA-based leading edge gene feature method; GPF: GSEA-based pathway feature method; SPF: SVM-based pathway feature method; RPF: Randomly selected pathway feature method; SKG: Single KEGG pathway gene method; SG: All single gene method.

## Competing interests

The authors declare that they have no competing interests related to this paper.

## Authors’ contributions

MK and CD initiated the project, SK implemented and tested the algorithm, MK and CD provided guidance for the project, SK and MK wrote the paper, and SK, MK, and CD contributed to the work through discussion and editing of the paper. All authors read and approved the final manuscript.

## Supplementary Material

Additional file 1 **Table S1.** Invariant leading edge gene/Fisher selected genes between data sets. Click here for file

Additional file 2 **Table S2.** Enriched pathways discussed in literature references. Click here for file
