# Supplementary material for: Pathway-based classification of cancer subtypes
Source: Biol Direct. 2012 Jul 3;7:21. doi: 10.1186/1745-6150-7-21 (PMC3485163; doi:10.1186/1745-6150-7-21)
Supplement: Additional file 1 — Table S1. Invariant leading edge gene/Fisher selected genes between data sets. [file 1745-6150-7-21-S1.pdf]

Additional file 1.

Table S1. (a) Invariant leading edge/Fisher selected genes between the Wang/van de Vijver breast cancer metastasis datasets. Genes listed are in the intersection of the leading edge genes and the Fisher selected genes in the Wang dataset, which are simultaneously in the intersection of same two groups of genes relative to the the van de Vijver dataset.

(b) Invariant leading edge/Fisher selected genes between the UNC and BI ovarian survival datasets. Genes listed are in the intersection of the leading edge genes and the Fisher selected genes in the UNC dataset, and simultaneously in the intersection of the same two groups of genes relative to the BI dataset. The enriched pathway through which each gene is selected is listed below the gene, and a summary of the gene/pathway roles is listed. Red indicates a pathway enriched for metastatic breast cancer or short survival ovarian cancer, while blue indicates enriched pathways in non-metastatic breast cancer and long survival time of ovarian cancer.

a) Genes related with breast cancer metastasis between Wang and van de Vijver data sets (pathway colors: red for enrichment in metastatic cancers, black in non-metastatic cancers)

Gene symbol<br>Pathway information	Full name	Summary
CCNB2<br>CELL_CYCLE	Cyclin B2	Involved with proliferating cells through binding to and activating p34[1]
PSMA7<br>PROTEASOME	Proteasome subunit, alpha-type, 7	Differentially expressed and associated with metastasis to the liver in colorectal cancer [2]
CCNE2<br>CELL_CYCLE	Cyclin E2	Induced oncogene, elevated in tumor-derived cells[3]
PTTG1<br>CELL_CYCLE	Pituitary tumor-transforming gene1	Prognostic marker for breast cancer[4] and colon cancer[5]. Overexpressed in most cancers; its levels correlate with tumor development and size[6,7].
TPI1<br>FRUCTOSE_AND_<br>MANNOSE_METABOLISM	Triosephosphate isomerase 1	anti-drug resistance agent in gastric cancer cells[8]
RRM2<br>PYRIMIDINE_METABOLISM	Ribonucleotide reductase, M2	Required for cell division, also found to speed up cell proliferation and progression after knockdown; overexpressed in pancreatic adenocarcinoma cells[9]
MAD2L1	Mitotic arrest-deficient 2, S. Cerevisiae, homolog-like 1	Overexpressed in breast cancer and ovarian cancer; MAD2 expression significant in mitotic

CELL_CYCLE		checkpoint control in ovarian cancer cells,[10,11,12] Dysfunction may lead to malignancy or cell degeneration. A checkpoint in turmorigenesis, overexpressed in several tumor types; targets E2F [13].
BUB1B<br>CELL_CYCLE	Budding uninhibited by benzimidazoles1, S. Cerevisiae homolog of beta	Mutations identified in somatic and germline transition of colorectal cancer cell lines; overexpressed 80% in breast cancer tissues, involved in spindle damage checkpoint[10]
SQLE<br>BIOSYNTHESIS_OF_STEROIDS	Squalene epoxidase	Overexpressed in breast cancer and significantly inversely related to distant metastasis-free survival in stage I/II breast cancer[14]
E2F1<br>CELL_CYCLE	E2F transcription factor 1	Levels strongly related with breast cancer outcome[15], melanoma progression and metastasis[16]
NP<br>PYRIMIDINE_METABOLISM	Nucleoside phosphorylase	
PSMB5<br>PROTEASOME	Proteasome subunit, beta type5	
TSTA3<br>FRUCTOSE_AND_MANNOSE_ METABOLISM	Tissue specific transplantation antigen P35B	Conserved gene for several breast cancer subtypes [17]

b) Genes related with ovarian cancer survival time (pathway colors: red for enrichment in metastatic cancers, blue in non-metastatic cancers)

Gene symbol<br>Pathway information	Full name	Summary
POLR1D<br>RNA_POLYMERASE	Polymerase (RNA) II (DNA directed) polypeptide D	Discriminator for late stage colon cancer[18]
ID4<br>[22]	Inhibitor of DNA binding 4	Regulator of BRCA1 expression[19]; putative tumor suppressor in human leukemia[20]; inhibitor of BRCA1 in breast cancer ovarian cancer[21]
EDAR<br>CYTOKINE_CYTOKINE_ RECEPTOR_INTERACTION	Ectodysplasin A receptor	Encodes a memeber of the tumor necrosis factor receptor family (Entrez)
BMPR2<br>TGF_BETA_SIGNALING_ PATHWAY	Bone morphogenetic protien receptor, type II	Pulmonary arterial hypertension[23]
HLA-DOA<br>CELL_ADHESION_MOLECULES	Major histcompatibility complex, class II, DO alpha	Related with B-cell malignancies [24]

TYPE_1_DIABETES_MELLITUS		
DPYSL3<br>Ovarian cancer module	Dihydropyrimidinase-like 3	Related with juvenile pilocytic astrocytomas (JPA), brain tumor grade 1[25]
ANXA4<br>Ovarian cance module	Annexin A4	Related with clear cell ovarian tumor chemotherapy resistance[26]
CXCL9<br>CYTOKINE_CYTOKINE_ RECEPTOR_INTERACTION	Chemoline (C-X-C motif) ligand 9	breast cancer-related gene[22]
MYLK<br>Ovarian cancer module	Myosin light chain kinase (MLCK)	Inhibiting myosin light chain kinase retards the growth of mammary and prostate cancer cells[27]; breast cancer related[28]
FBXL7<br>Ovarian cancer module	F-box and leucine-rich repeat protein 7	Breast cancer related [29]
TBL1X<br>WNT_SIGNALING_PATHWAY	Transfucin (beta)-like 1X-linked	Related to endometrial carcinogenesis[30]
