# Supplementary material for: Pathway-based classification of cancer subtypes
Source: Biol Direct. 2012 Jul 3;7:21. doi: 10.1186/1745-6150-7-21 (PMC3485163; doi:10.1186/1745-6150-7-21)
Supplement: Additional file 2 — Table S2. Enriched pathways discussed in literature references. [file 1745-6150-7-21-S2.pdf]

Additional file 2.

Table S2. Remaining common enriched pathways between two cohorts (cancer-related pathways are listed in table II of the main paper). First part of the table lists the common enriched pathways between the BI and UNC data sets, associated with ovarian cancer survival. Second part of the table presents common enriched pathways associated with metastatic breast cancer between the van de Vijver and Wang data sets. Red coloring represents enriched pathways in short (versus long) survival for ovarian cancer, and for metastasis in breast cancer, and blue coloring represents enriched pathways in long survival and non-metastasis, respectively. Asterisks (*) indicate pathways also distinguished in the Dressman et al [31] study.

Common pathways for two ovarian survival data sets		
Enriched pathway		Function of pathway
Enriched Pathways in long survival time	**HSA04940_TYPE_1_DIABETES_MELLITUS**	Includes human leukocyte antigen (HLA) genes, related to immune system function and protection against cancer,
	HSA04514_CELL_ADHESION_MOLECULES* [32]	Known to interfere with cellular detachment and cancer metastasis, related to cancer invasion and metastasis.
	HSA05217_BASAL_CELL_CARCINOMA	
	HSA04916_MELANOGENESIS	
	HSA03030_DNA_POLYMERASE[33]	Functions to replicate DNA, targeting DNA damage agent which cause cell cycle arrest[33] .
	HSA04310_WNT_SIGNALING_PATHWAY*	Includs oncogenes and tumor suppressors[34];significantly deregulated in ovarian cancer[35]

	**HSA04340_HEDGEHOG_SIGNALING_ PATHWAY***	Crucial role in tumorigenesis[36]
	HSA04612_ANTIGEN_PROCESSING_AND_ PRESENTATION*	Biological process for preparing antigens for presentation to immune system cells.
	HSA04350_TGF_BETA_SIGNALING_PATHWAY*	Affects tumorigenesis, either negatively or positively[37].
	HSA00251_GLUTAMATE_METABOLISM	
	**HSA04060_CYTOKINE_CYTOKINE_ RECEPTOR_INTERACTION***	Involves cytokines which facilitate invasion and metastasis and also function to inhibit tumor progression[38] .
	**Ovarian_cancer module (Stanford)**	Curated ovarian cancer-related gene set from Stanford study [39]
Enriched pathways in short survival time	HSA03020_RNA_POLYMERASE	Elevated levels of RNA polymerase products appear in tumor cells[40] .
	HSA01032_GLYCAN_STRUCTURES_ DEGRADATION	N-linked glycan is generated by tumor cells at high levels and plays an important role in cell-cell communication.
	HSA00220_UREA_CYCLE_AND_METABOLISM_ OF_AMINO_GROUPS	
	HSA01031_GLYCAN_STRUCTURES_ BIOSYNTHESIS_2*	
	HSA00903_LIMONENE_AND_PINENE_ DEGRADATION	
	HSA00920_SULFUR_METABOLISM	Constituent of a number of essential cellular processes such as biosynthesis or transfer of electrons
	Ovarian_Schwarts	Curated ovarian gene set distinguishes clear cell from other poor-prognosis ovarian cancers (stage III and stage IV)[41]; one of 5 additional curated ovarian-related gene sets used in this paper
	Ovarian_Schaner	Clear cell carcinoma predictor from other ovarian epithelial cancers[42];one of 5 additional curated ovarian-related gene sets.

Common pathways for metastasis & non-metastasis between van de Vijver and Wang data sets	
Enriched pathway	Function of pathway

Enriched pathways in non-metastasis	**HSA04940_TYPE_1_DIABETES_MELLITUS**	Includes human leukocyte antigen (HLA), related with immune system function to protect against cancer and mediate autoimmune disease.
	HSA04640_HEMATOPOIETIC_CELL_LINEAGE	Crucial function of self-renewal in both stem cells and cancer cells [43]
	HSA04610_COMPLEMENT_AND_ COAGULATION_CASCADES	Inhibits lung cancer metastasis in animal studies[44]; part of innate immune system and an effector of antibody-mediated immunity [45].
	**HSA04060_CYTOKINE_CYTOKINE_ RECEPTOR_INTERACTION**	Cancer cells respond cytokines which facilitate invasion and metastasis and also function to inhibit tumor progression[38]
	**HSA04340_HEDGEHOG_SIGNALING_ PATHWAY**	Crucial role in tumorigenesis [36] [46]
Enriched pathways in Metastasis	HSA00051_FRUCTOSE_AND_MANNOSE_ METABOLISM	
	HSA04110_CELL_CYCLE (cancer)	Related with cell growth and cell death
	HSA00100_BIOSYNTHESIS_OF_STEROIDS (cancer)	Found in metastatic tissue [47], and related with feminizing syndromes [48]
	HSA03050_PROTEASOME	Degradation of damaged and unneeded proteins
	HSA05110_CHOLERA_INFECTION	
	HSA00240_PYRIMIDINE_METABOLISM	
	HSA00970_AMINOACYL_TRNA_ BIOSYNTHESIS	

References

1. Brandeis M, Rosewell I, Carrington M, Crompton T, Jacobs MA, et al. (1998) Cyclin B2-null mice develop normally and are fertile whereas cyclin B1-null mice die in utero. Proc Natl Acad Sci U S A 95: 4344-4349.
2. Hu XT, Chen W, Zhang FB, Shi QL, Hu JB, et al. (2009) Depletion of the proteasome subunit PSMA7 inhibits colorectal cancer cell tumorigenicity and migration. Oncol Rep 22: 1247-1252.
3. Wu Z, Cho H, Hampton GM, Theodorescu D (2009) Cdc6 and cyclin E2 are PTEN-regulated genes associated with human prostate cancer metastasis. Neoplasia 11: 66-76.
4. Solbach C, Roller M, Fellbaum C, Nicoletti M, Kaufmann M (2004) PTTG mRNA expression in primary breast cancer: a prognostic marker for lymph node invasion and tumor recurrence. Breast 13: 80-81.
5. Heaney AP, Singson R, McCabe CJ, Nelson V, Nakashima M, et al. (2000) Expression of pituitary-tumour transforming gene in colorectal tumours. Lancet 355: 716-719.
6. Saez C, Japon MA, Ramos-Morales F, Romero F, Segura DI, et al. (1999) hpttg is over-expressed in pituitary adenomas and other primary epithelial neoplasias. Oncogene 18: 5473-5476.
7. McCabe CJ, Khaira JS, Boelaert K, Heaney AP, Tannahill LA, et al. (2003) Expression of pituitary tumour transforming gene (PTTG) and fibroblast growth factor-2 (FGF-2) in human pituitary adenomas: relationships to clinical tumour behaviour. Clin Endocrinol (Oxf) 58: 141-150.
8. Wang X, Lu Y, Yang J, Shi Y, Lan M, et al. (2008) Identification of triosephosphate isomerase as an anti-drug resistance agent in human gastric cancer cells using functional proteomic analysis. J Cancer Res Clin Oncol 134: 995-1003.
9. Duxbury MS, Ito H, Zinner MJ, Ashley SW, Whang EE (2004) RNA interference targeting the M2 subunit of ribonucleotide reductase enhances pancreatic adenocarcinoma chemosensitivity to gemcitabine. Oncogene 23: 1539-1548.

10. Yuan B, Xu Y, Woo JH, Wang Y, Bae YK, et al. (2006) Increased expression of mitotic checkpoint genes in breast cancer cells with chromosomal instability. Clinical Cancer Research 12: 405-410.
11. Wang Z, Zhao B, Xu Y, Wan Y, Bu D, et al. (2002) [Genealogical research of hereditary nonpolyposis colorectal cancer]. Zhonghua Wai Ke Za Zhi 40: 411-413.
12. Wang X, Jin DY, Ng RW, Feng H, Wong YC, et al. (2002) Significance of MAD2 expression to mitotic checkpoint control in ovarian cancer cells. Cancer Research 62: 1662-1668.
13. Percy MJ, Myrie KA, Neeley CK, Azim JN, Ethier SP, et al. (2000) Expression and mutational analyses of the human MAD2L1 gene in breast cancer cells. Genes Chromosomes Cancer 29: 356-362.
14. Helms MW, Kemming D, Pospisil H, Vogt U, Buerger H, et al. (2008) Squalene epoxidase, located on chromosome 8q24.1, is upregulated in 8q+ breast cancer and indicates poor clinical outcome in stage I and II disease. Br J Cancer 99: 774-780.
15. Vuaroqueaux V, Urban P, Labuhn M, Delorenzi M, Wirapati P, et al. (2007) Low E2F1 transcript levels are a strong determinant of favorable breast cancer outcome. Breast Cancer Research 9: R33.
16. Alla V, Engelmann D, Niemetz A, Pahnke J, Schmidt A, et al. (2010) E2F1 in melanoma progression and metastasis. J Natl Cancer Inst 102: 127-133.
17. Yu K, Lee CH, Tan PH, Tan P (2004) Conservation of breast cancer molecular subtypes and transcriptional patterns of tumor progression across distinct ethnic populations. Clinical Cancer Research 10: 5508-5517.
18. Nibbe RK, Markowitz S, Myeroff L, Ewing R, Chance MR (2009) Discovery and scoring of protein interaction subnetworks discriminative of late stage human colon cancer. Mol Cell Proteomics 8: 827-845.
19. Beger C, Pierce LN, Kruger M, Marcusson EG, Robbins JM, et al. (2001) Identification of Id4 as a regulator of BRCA1 expression by using a ribozyme-library-based inverse genomics approach. Proc Natl Acad Sci U S A 98: 130-135.
20. Yu L, Liu C, Vandeusen J, Becknell B, Dai Z, et al. (2005) Global assessment of promoter methylation in a mouse model of cancer identifies ID4 as a putative tumor-suppressor gene in human leukemia. Nature Genetics 37: 265-274.
21. Welcsh PL, King MC (2001) BRCA1 and BRCA2 and the genetics of breast and ovarian cancer. Hum Mol Genet 10: 705-713.
22. Ruiz-Garcia E, Scott V, Machavoine C, Bidart JM, Lacroix L, et al. (2010) Gene expression profiling identifies Fibronectin 1 and CXCL9 as candidate biomarkers for breast cancer screening. Br J Cancer 102: 462-468.
23. Cogan JD, Pauciulo MW, Batchman AP, Prince MA, Robbins IM, et al. (2006) High frequency of BMPR2 exonic deletions/duplications in familial pulmonary arterial hypertension. Am J Respir Crit Care Med 174: 590-598.
24. van Lith M, van Ham M, Neefjes J (2002) Novel polymorphisms in HLA-DOA and HLA-DOB in B-cell malignancies. Immunogenetics 54: 591-595.
25. Wong KK, Chang YM, Tsang YT, Perlaky L, Su J, et al. (2005) Expression analysis of juvenile pilocytic astrocytomas by oligonucleotide microarray reveals two potential subgroups. Cancer Research 65: 76-84.
26. Kim A, Enomoto T, Serada S, Ueda Y, Takahashi T, et al. (2009) Enhanced expression of Annexin A4 in clear cell carcinoma of the ovary and its association with chemoresistance to carboplatin. International Journal of Cancer 125: 2316-2322.
27. Gu LZ, Hu WY, Antic N, Mehta R, Turner JR, et al. (2006) Inhibiting myosin light chain kinase retards the growth of mammary and prostate cancer cells. Eur J Cancer 42: 948-957.
28. Khuon S, Liang L, Dettman RW, Sporn PH, Wysolmerski RB, et al. (2010) Myosin light chain kinase mediates transcellular intravasation of breast cancer cells through the underlying endothelial cells: a three-dimensional FRET study. J Cell Sci 123: 431-440.
29. Wang X, Pankratz VS, Fredericksen Z, Tarrell R, Karaus M, et al. (2010) Common variants associated with breast cancer in genome-wide association studies are modifiers of breast cancer risk in BRCA1 and BRCA2 mutation carriers. Hum Mol Genet 19: 2886-2897.
30. Boren T, Xiong Y, Hakam A, Wenham R, Apte S, et al. (2008) MicroRNAs and their target messenger RNAs associated with endometrial carcinogenesis. Gynecologic Oncology 110: 206-215.

31. Dressman HK, Berchuck A, Chan G, Zhai J, Bild A, et al. (2007) An integrated genomic-based approach to individualized treatment of patients with advanced-stage ovarian cancer. Journal of Clinical Oncology 25: 517-525.
32. Behrens J (1993) The role of cell adhesion molecules in cancer invasion and metastasis. Breast Cancer Res Treat 24: 175-184.
33. Helleday T, Petermann E, Lundin C, Hodgson B, Sharma RA (2008) DNA repair pathways as targets for cancer therapy. Nat Rev Cancer 8: 193-204.
34. Polakis P (2000) Wnt signaling and cancer. Genes Dev 14: 1837-1851.
35. Gatcliffe TA, Monk BJ, Planutis K, Holcombe RF (2008) Wnt signaling in ovarian tumorigenesis. International Journal of Gynecological Cancer 18: 954-962.
36. Rubin LL, de Sauvage FJ (2006) Targeting the Hedgehog pathway in cancer. Nat Rev Drug Discov 5: 1026-1033.
37. Wakefield LM, Roberts AB (2002) TGF-beta signaling: positive and negative effects on tumorigenesis. Curr Opin Genet Dev 12: 22-29.
38. Dranoff G (2004) Cytokines in cancer pathogenesis and cancer therapy. Nat Rev Cancer 4: 11-22.
39. Segal E, Friedman N, Koller D, Regev A (2004) A module map showing conditional activity of expression modules in cancer. Nature Genetics 36: 1090-1098.
40. White RJ (2004) RNA polymerase III transcription and cancer. Oncogene 23: 3208-3216.
41. Schwartz DR, Kardia SL, Shedden KA, Kuick R, Michailidis G, et al. (2002) Gene expression in ovarian cancer reflects both morphology and biological behavior, distinguishing clear cell from other poor-prognosis ovarian carcinomas. Cancer Research 62: 4722-4729.
42. Schaner ME, Ross DT, Ciaravino G, Sorlie T, Troyanskaya O, et al. (2003) Gene expression patterns in ovarian carcinomas. Molecular Biology of the Cell 14: 4376-4386.
43. Reya T, Morrison SJ, Clarke MF, Weissman IL (2001) Stem cells, cancer, and cancer stem cells. Nature 414: 105-111.
44. Bruggemann LW, Versteeg HH, Niers TM, Reitsma PH, Spek CA (2008) Experimental melanoma metastasis in lungs of mice with congenital coagulation disorders. J Cell Mol Med 12: 2622-2627.
45. Markiewski MM, Nilsson B, Ekdahl KN, Mollnes TE, Lambris JD (2007) Complement and coagulation: strangers or partners in crime? Trends Immunol 28: 184-192.
46. Taipale J, Beachy PA (2001) The Hedgehog and Wnt signalling pathways in cancer. Nature 411: 349-354.
47. Touitou Y, Bogdan A, Auzeby A (1983) Experimental evidence for biosynthesis of steroids in metastatic tissue originating from a primitive adrenocortical carcinoma. Int J Biochem 15: 571-573.
48. Dorfman RI, Sharma DC, Southren AL, Gabrilove JL (1965) Biosynthesis of steroids in various tissues related to feminizing syndromes. Cancer Research 25: 1125-1128.
